# Meta-analysis of the association between angiotensin pathway inhibitors and COVID-19 severity and mortality

**DOI:** 10.1186/s13643-021-01802-6

**Published:** 2021-09-07

**Authors:** Malindu E. Fernando, Aaron Drovandi, Jonathan Golledge

**Affiliations:** 1 Queensland Research Centre for Peripheral Vascular Disease, College of Medicine and Dentistry, James Cook University, Townsville, Queensland 4811 Australia; 2 Department of Vascular and Endovascular Surgery, Townsville University Hospital, Townsville, Queensland Australia; 3 Australian Institute of Tropical Health and Medicine, James Cook University, Townsville, Queensland Australia

**Keywords:** 2019 novel coronavirus disease, Angiotensin-converting enzyme inhibitors, Angiotensin receptor blockers, COVID-19

## Abstract

**Background:**

Conflicting findings and the analysis of unpublished and retracted data have led to controversy on the safety of angiotensin-converting enzyme inhibitors and angiotensin receptor blockers in people with COVID-19 infection. This meta-analysis examined the association of prescription of angiotensin-converting enzyme inhibitors (ACEI) and angiotensin receptor blockers (ARB) with the outcome from COVID-19.

**Methods:**

A systematic search was conducted to find published studies that reported the outcome of COVID-19 in relation to prescription of ACEI or ARB. Two authors (MF and AD) independently screened and extracted data and assessed study quality and strength of association using standardised tools. The endpoints for the meta-analyses were severe or critical disease outcome and mortality based on standardised criteria.

**Results:**

Twenty-six studies including 8389 people prescribed ACEI or ARB and 20,989 people not prescribed these medications were included. The quality of studies varied, and the overall strength of association was poor with a high risk of confounding bias. Patients prescribed ACEI or ARB had a greater prevalence of risk factors. Meta-analysis found an association between prescription of ACEI or ARB with severe or critical disease outcome (risk ratio, RR, 1.23, 95% confidence interval, CI, 1.06 to 1.42, *p* = 0.006, *I*^2^ = 88%) but this association was lost in sensitivity analyses. There was no association between ACEI or ARB prescription and mortality (RR 1.18, 95% CI 0.92 to 1.50, *p* = 0.19, *I*^2^ = 82%).

**Conclusions:**

This meta-analysis suggests that people prescribed ACEI or ARB more commonly had severe or critical disease outcome, but not mortality, in published cohorts of patients diagnosed with COVID-19. This finding is most likely due to a greater prevalence of risk factors in these patients rather than due to exposure to angiotensin pathway inhibitors.

**Supplementary Information:**

The online version contains supplementary material available at 10.1186/s13643-021-01802-6.

## Introduction

The SARS-CoV-2 virus, which is responsible for COVID-19, is believed to bind to host cells via angiotensin-converting enzyme 2 (ACE2) [1]. ACE2 expression in some experimental models is altered (both up and downregulation has been reported) by the commonly used antihypertensive drugs angiotensin-converting enzyme inhibitors (ACEI) and angiotensin receptor blockers (ARB) [2, 3]. Meta-analyses suggest that people with a history of hypertension have poor outcomes from COVID-19 [4, 5] that could conceivably be due to the prescription of ACEI or ARBs [6].

A number of previous meta-analyses and reviews have explored the association of ACEI and ARB exposure with the outcome of COVID-19 but the findings have been inconsistent [7–11]. Some have reported no significant association between ACEI or ARB prescription and outcome [7–9], whilst others have found reduced mortality in patients prescribed these medications [10, 11]. These previous reviews have had a number of deficiencies, including failure to assess the quality of the included studies and limited examination of the strength of associations identified. Previous analyses have also included unpublished and later retracted studies, leading to concerns about the validity of findings [12]. Furthermore, many new relevant studies have since been published. The aim of this systematic review was to provide an up-to-date and robust assessment of the association of ACEI and ARB exposure with the outcome of COVID-19.

## Material and methods

### Search strategy

This systematic review and meta-analysis was conducted according to the Preferred Reporting Items for Systematic reviews and Meta-Analyses (PRISMA) statement and the Meta-analysis Of Observational Studies in Epidemiology (MOOSE) reporting standards [13, 14]. The final search for original studies was performed on the 19th of June 2020 using multiple databases [MEDLINE via OvidSP, and PubMed]. The search string is shown in S**1****: Text Box 1**. Reference lists of identified publications were hand searched to identify other potentially eligible studies.

### Inclusion of studies

Prospective or retrospective cohort and case-control studies reporting the outcome from COVID-19 in relation to ACEI and/or ARB prescription were eligible for inclusion. Animal studies, reviews and editorials were excluded. Only articles published in English were included and there were no restrictions on publication date. Titles and abstracts of publications identified were screened by two authors (MF, AD) to find studies meeting the eligibility criteria. The full texts of publications that appeared relevant were reviewed by both authors and a decision regarding inclusion made by consensus with the third author (JG).

### Definitions of COVID-19, risks factors, drug exposure and outcomes

COVID-19 diagnosis was based on World Health Organisation (WHO) interim laboratory testing for COVID-19 criteria [15]. Hypertension, diabetes, ischaemic heart disease (IHD), chronic obstructive pulmonary disease (COPD), congestive cardiac failure (CCF) and chronic kidney disease (CKD) were defined by prior diagnosis on electronic medical records as per the international statistical classification of diseases and related health problems version 10 (ICD-10) [16]. Medications, including ACEI or ARB, were based on record of drug prescription at the time of hospital admission. The primary outcomes were severe or critical disease and mortality according to a report of the WHO-China Joint Mission on COVID-19 [17]. Severe disease was defined as the presence of tachypnoea (≥ 30 breaths/min), oxygen saturation ≤ 93% at rest, arterial oxygen tension (PaO_2_) over inspiratory oxygen fraction (FIO_2_) PaO2/FiO2 ratio of < 300 mmHg, or a clinical diagnosis of ARDS or prolonged hospitalisation (≥ 10 days) [17]. Critical disease was defined as people with respiratory failure requiring mechanical ventilation, shock or other organ failure that requires intensive care. A severe or critical disease outcome of COVID-19 was defined to include both severe and critical disease definitions or death. Mortality was defined as any in-hospital death where COVID-19 was thought to have contributed, as per the WHO guidelines [18]. Exposure to ACEI and/or ARB prescription was defined as a prescription of these medication classes preceding a diagnosis of COVID-19 irrespective of continued medication use at the time of hospitalisation.

### Data extraction

Data extracted included the number of primary outcome events in relation to prescription of ACEI/ARB (exposure), the country where the study took place, study design, sample size, age, sex, hypertension, prescribed anti-hypertensive medications, comorbidities, systolic and diastolic blood pressure (mmHg) and biochemical data at admission [potassium (mmol/l), creatinine (μ mol/L), estimated glomerular filtrated rate (ml/min per 1.73 m^2^) and C-reactive protein (CRP) (mg/L)]. All data were independently extracted by two authors (MF, AD) using a standardised template and inconsistencies were resolved through discussion with a third author (JG). The definitions used by studies are reported in **S1****: Supp Table 1**. To obtain missing data or clarify any discrepancies, corresponding authors of all studies were contacted via email, of whom 13 responded. Five authors replied with additional data [19–23], and eight authors responded but could not provide additional data [21, 24–30]. A number of publications reported potentially overlapping data through utilising the same hospitals during the same time period [11, 19, 31–33] and therefore sensitivity analyses were performed whilst excluding and including these studies.

### Quality assessment

Two assessors (MF, AD) independently evaluated the quality of studies using a modified version of the Risk Of Bias In Non-randomised Studies of Interventions tool [34]. The items assessed included participant selection bias, information bias of study outcomes, definition of exposure of ACEI/ARB prescription, reporting bias due to missing data and the risk of confounding [34]. Any inconsistencies were resolved through discussion until consensus was reached. Each item (10 questions) was assessed as yes, no or not reported. A “yes” was scored as 1 and a “no” or “not reported” as 0. The scoring of all items was then summed and presented as a percentage of the total possible score of 10. The final agreed and individual quality scores and degree of agreement were reported.

### Evaluating the strength of association

Two assessors (MF, AD) independently evaluated the strength of association between ACEI/ARB prescription and COVID-19 outcome using relevant components of the Sir Bradford Hill Criteria: dose response relationship, temporal sequence and protopathic bias (timing and duration of ACEI/ARB prescription), biological plausibility and specificity of exposure and outcome [35].

### Statistical analysis

The main meta-analysis examined the association between exposure to ACEI or ARBs (as a combined group) and outcome from COVID-19. Subgroup analyses were performed to examine the associations of ACEI prescription alone or ARB prescription alone with outcome. Leave-one-out sensitivity analyses were performed to assess the contribution of each study to the pooled estimates by excluding individual studies one at a time and recalculating the pooled estimates [36]. Sensitivity analyses were also performed focusing on people with a history of hypertension alone, excluding potential overlapping cohorts and focusing on studies from individual continents (Asia, USA or Europe) and high-quality investigations (defined as quality assessment scores ≥ 90%). All meta-analyses were performed using Mantel-Haenszel’s statistical method and random effect models anticipating substantial heterogeneity [37]. The results were reported as risk ratios (RR) and 95% confidence intervals (CI). All statistical tests were two-sided and a *p* value < 0.05 was considered significant. Heterogeneity was assessed using the I^2^ statistic values (interpreted as 0 to 49%: low, 50 to 74%: moderate and 75 to 100%: high) [38]. Publication bias was assessed by funnel plots comparing the summary estimate of each study and its precision (1/standard error) [36]. All analyses were conducted using Review Manager 5 (RevMan 5) version 5.3. (Copenhagen: Nordic Cochrane Centre, The Cochrane Collaboration, 2014).

## Results

### Search results

**Fig. 1:**
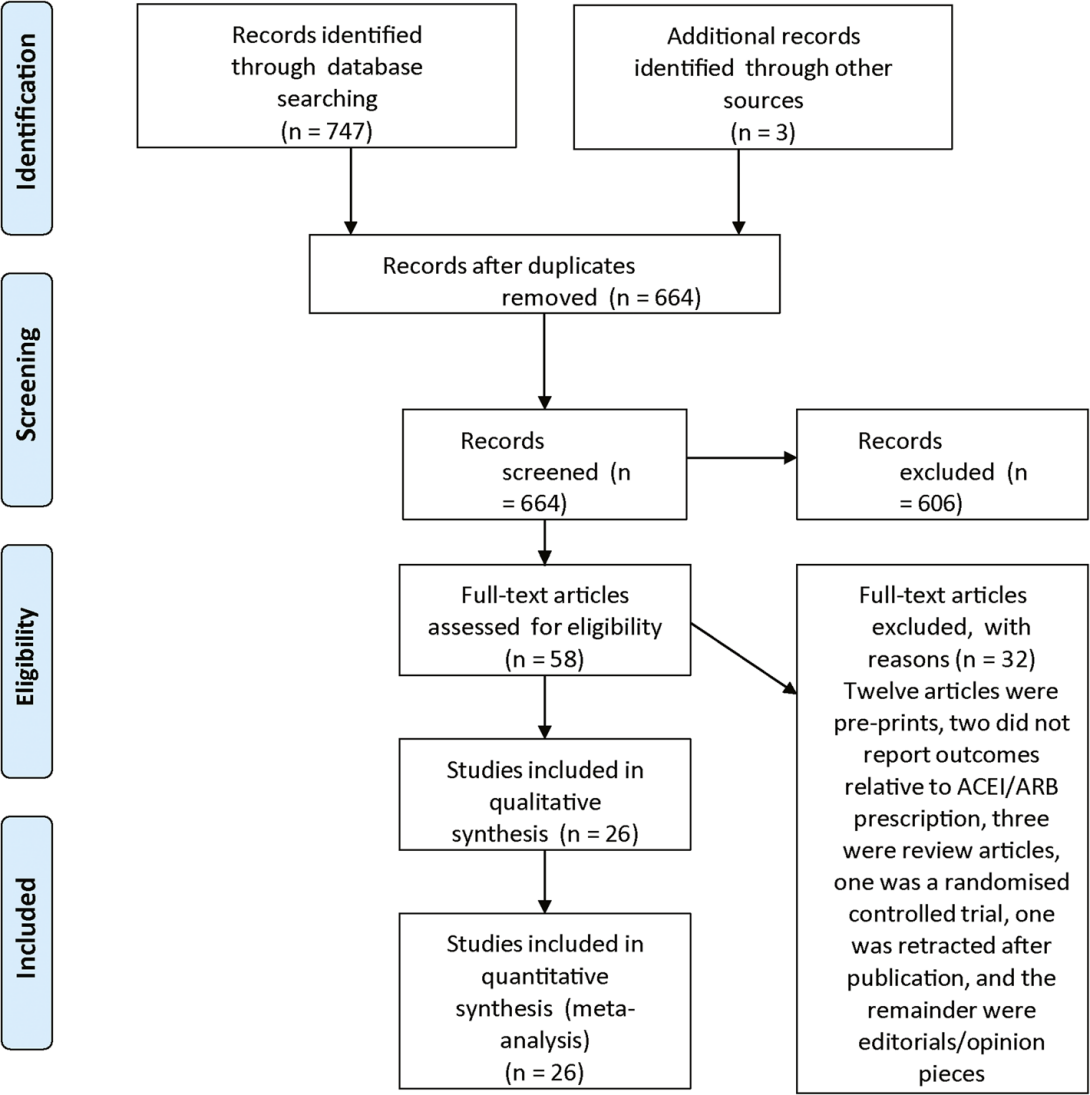
PRISMA flow diagram detailing the search results on the 19th June 2020

Twenty-six studies from 664 identified articles met the eligibility criteria and were included (Fig. 1) [19–33, 39–49].

### Cohort characteristics

Thirteen studies were from China, five from Italy, three from the USA and one each from Denmark, Spain, Korea, the UK and France. A total of 29,378 out of 44,454 people diagnosed with COVID-19 comprising 8389 people who were prescribed ACEI/ARB and 20,989 people not prescribed ACEI/ARB were included in the meta-analysis (**S1****: Supp Table 2-3**). The characteristics of participants stratified by the prescription of ACEI/ARB are reported in Table 1. The weighted average age of people prescribed ACEI/ARB was higher than the control group (70 years vs. 56 years). Participants in the ACEI/ARB group were more likely to be male (58% vs. 50%) and have hypertension (92% vs. 39%), diabetes (30% vs. 11%), IHD (21% vs. 7%), CCF (14% vs. 3%). CKD (10% vs. 3%) and COPD (12% vs. 7%) than those not prescribed these drugs. Additional data including blood pressure, C-reactive protein, incidence of severe or critical disease and mortality according to ACEI/ARB and differences in the inpatient use of steroids and anti-viral medications stratified by groups is reported in S**1****: Supp Table 2-6.**

**Table 1:** Study design and characteristics of included patients according to angiotensin converting enzyme inhibitor or angiotensin receptor blocker prescription

Study	Country	Study design	Characteristics of people prescribed ARBs/ACEI (***n*** = 8389)									Characteristics of people not prescribed ARBs/ACEI (***n*** = 20989)								
			*N*	Age	Males	HTN	DM	IHD	CCF	CKD	COPD	*N*	Age	Males	HTN	DM	IHD	CCF	CKD	COPD
Feng et al. (2020) [39]	China	Retrospective cohort	33	NR	NR	33 (100%)	NR	NR	NR	NR	NR	443	NR	NR	80 (18%)	NR	NR	NR	NR	NR
Gao et al. (2020) [40]	China	Retrospective cohort	183	63 (SD 11)	104 (57%)	183 (100%)	55 (28%)	32 (16%)	1 (1%)	2 (1%)	1 (1%)	2694	NR	1366 (51%)	667 (25%)	332 (12%)	201 (7%)	22 (1%)	27 (1%)	30 (1%)
Guo et al. (2020) [41]	China	Retrospective cohort	19	NR	NR	NR	NR	NR	NR	NR	NR	168	NR	NR	NR	NR	NR	NR	NR	NR
Hu et al. (2020) [42]	China	Retrospective cohort	65	56 (48–64)	40 (62%)	65 (100%)	16 (25%)	2 (3%)		4 (6%)	1 (2%)	819	NR	415 (51%)	84 (10%)	49 (6%)	13 (2%)			
Huang et al. (2020) [19]	China	Retrospective cohort	20	53 (SD 13)	10 (50%)	20 (100%)	0	0	NR	NR	1 (5%)	30	67 (SD 13)	17 (57%)	30 (100%)	4 (13%)	1 (3%)	NR	NR	0
Li & Wang et al. (2020) [43]	China	Retrospective cohort	115	65 (57–73)	68 59%)	115 (100%)	42 (37%)	27 (24%)	5 (4%)	13 (11%)	8 (7%)	247	67 (60–75)	121 (49%)	247 (100%)	85 (34%)	35 (14%)	5 (2%)	22 (9%)	10 (4%)
Li & Xu et al. (2020) [32]	China	Ambispective cohort	42	NR	NR	NR	NR	NR	NR	NR	NR	506	NR	NR	NR	NR	NR	NR	NR	NR
Meng et al. (2020) [20]	China	Retrospective cohort	17	64 (56–69)	8 (47%)	17 (100%)	2 (12%)	2 (12%)	NR	NR	NR	25	65 (55–68)	15 (60%)	25 (100%)	4 (16%)	6 (24%)	NR	NR	NR
Tan et al. (2020) [44]	China	Retrospective cohort	31	67 (62–70)	NR	31 (100%)	8 (26%)	5 (16%)	NR	4 (13%)	2 (6%)	69	68 (57–71)	NR	69 (100%)	20 (29%)	13 (19%)	NR	5 (7%)	7 (10%)
Wang et al. (2020) [33]	China	Retrospective cohort	62	NR	NR	NR	NR	NR	NR	NR	NR	282	NR	NR	NR	NR	NR	NR	NR	NR
Yang et al. (2020) [24]	China	Retrospective cohort	43	65 (57–72)	21 (49%)	43 (100%)	13 (30%)	7 (16%)		0	3 (7%)	208	NR	102 (49%)	83 (40%)	42 (20%)	28 (13%)			
Zhang et al. (2020) [31]	China	Retrospective cohort	188	64 (55–68)	100 (53%)	188 (100%)	44 (23%)	29 (15%)		7 (4%)	1 (1%)	940	64 (57–69)	503 (54%)	940 (100%)	118 (13%)	56 (6%)	NR	19 (2%)	3 (0%)
Zhou et al. (2020) [45]	China	Retrospective cohort	15	59 (SD 10)	9 (60%)	15 (100%)	NR	NR	NR	NR	NR	95	NR	51 (54%)	21 (22%)	NR	NR	NR	NR	NR
Fosbol et al. (2020) [25]	Denmark	Retrospective cohort	895	73 (61–81)	493 (55%)	634 (71%)	217 (24%)	193 (21%)	131 (15%)	67 (7%)	171 (19%)	3585	50 (37–65)	1651 (46%)	209 (6%)	194 (5%)	186 (5%)	112 (3%)	105 (3%)	463 (13%)
Liabeuf et al. (2020) [26]	France	Retrospective cohort	96	NR	NR	NR	NR	NR	NR	NR	NR	172	NR	NR	NR	NR	NR	NR	NR	NR
Cannata et al. (2020) [27]	Italy	Prospective cohort	173^#^	NR	NR	NR	NR	NR	NR	NR	NR	224	NR	NR	NR	NR	NR	NR	NR	NR
Conversano et al. (2020) [46]	Italy	Retrospective cohort	68	NR	NR	68 (100%)	NR	NR	NR	NR	NR	123	NR	NR	NR	NR	NR	NR	NR	NR
Felice et al. (2020) [21]	Italy	Retrospective cohort	82	NR	59 (72%)	82 (100%)	20 (24%)	29 (35%)		NR	7 (9%)	51	76 (SD 12)	27 (53%)	51 (100%)	14 (28%)	27 (53%)			
Mancia et al. (2020) [47]	Italy	Retrospective cohort	2896	NR	NR	NR	NR	NR	NR	NR	NR	3376	NR	NR	NR	NR	NR	NR	NR	NR
Tedeschi et al. (2020) [22]	Italy	Prospective cohort	175	NR	133 (76%)	175 (100%)	37 (21%)	71 (41%)	NR	NR	19 (11%)	136	NR	92 (68%)	136 (100%)	37 (27%)	60 (44%)	NR	NR	30 (22%)
Jung et al. (2020) [23]	Korea	Retrospective cohort	377	NR	NR	348 (92%)	NR	NR	NR	NR	NR	1577	NR	NR	194 (12%)	NR	NR	NR	NR	NR
de Abajo et al. (2020) [48]	Spain	Retrospective cohort	477	NR	NR	NR	NR	NR	NR	NR	NR	662	NR	NR	NR	NR	NR	NR	NR	NR
Bean at al. (2020) [49]	UK	Prospective cohort	399	73 (SD 13)	231 (58%)	339 (85%)	215 (54%)	83 (21%)	65 (16%)	108 (27%)	42 (11%)	801	65 (SD 18)	455 (57%)	306 (38%)	203 (25%)	77 (10%)	42 (5%)	98 (12%)	79 (10%)
Mehta et al. (2020) [28]	USA	Retrospective cohort	212	NR	NR	NR	NR	NR	NR	NR	NR	1523	NR	NR	NR	NR	NR	NR	NR	NR
Reynolds et al. (2020) [29]	USA	Retrospective study	1293	NR	NR	1293 (100%)	NR	NR	NR	NR	NR	1280	NR	NR	1280 (100%)	NR	NR	NR	NR	NR
Richardson et al. (2020) [30]	USA	Prospective cohort	413^#^	NR	NR	413 (100%)	NR	NR	NR	NR	NR	953	NR	NR	953 (100%)	NR	NR	NR	NR	NR
Weighted mean/%			**–**	**70**	**58%**	**92%**	**30%**	**21%**	**14%**	**10%**	**12%**	**-**	**56**	**50%**	**39%**	**11%**	**7%**	**3%**	**3%**	**7%**

Legend: Data are presented as number/total (percentage) or median (interquartile range) except where highlighted as mean (± SD). All values were rounded to the nearest integer. *NR* not reported, *UK* United Kingdom, *USA* United States of America. *N* number, *ACEI* angiotensin-converting enzyme inhibitor, *ARB* angiotensin-2 receptor blocker, *HTN* hypertension, DM diabetes mellitus, IHD ischemic heart disease/coronary artery disease, CCF congestive cardiac failure, COPD chronic obstructive pulmonary disease, *CKD* chronic kidney disease. The weighted average age was calculated using the average median or mean age reported in studies. In Li and Wang et al., outcomes were only reported for people with hypertension prescribed ACEI/ARB and as the use of ACEI/ARB could not be excluded in people without hypertension. In Liabeuf et al., a total of 499 local patients tested positive for SARS-CoV-2 and of these, 231 were not hospitalised. In Jung et al., amongst the 5179 patients with COVID-19, hospitalisation was observed for 1954 people and outcome data was only available for the hospitalised patients (38%). In Richardson et al., clinical data was available only for 46.2% of admitted patients and medication data was only available for (92%) of the included patients. In Reynolds et al. the pre-matching data was used and in Mehta et al. pre-matched data was unavailable. In Jung et al., only clinical outcome data for 1954 hospitalised patients were included. ^#^In Cannata et al, 56 out of 173 continued ACEI/ARB use and 117 discontinued at admission, and for Richardson et al., 227 out of 413 continued ACEI/ARB use during hospital admission. In Gao et al., 17/2694 were prescribed an ACEI/ARB but as outcome data was not available leave-one-out sensitivity analysis performed

### Study quality and strength of association

Quality scores from the two assessors are reported in **S1****: Supp Table 7**. There was agreement on 233 of the 260 (90%) assessments made. The final quality assessments reached after a consensus meeting are reported in Table 2. The overall quality of studies ranged from 20% (low) to 90% (high). All but eight articles reported on participant selection bias [19, 23, 25, 31, 33, 41, 45, 46]. All studies except three [22, 27, 44] reported the method of COVID-19 diagnosis (**S1****: Supp** **Table** **1****)**, but two studies [31, 46] included people with computed tomography-based diagnoses and two studies did not confirm all diagnoses with PCR [25, 31]. All but six studies provided outcome definitions for COVID-19 related mortality [20, 21, 28, 29, 43, 48]. Thirteen studies provided a clear or surrogate definition for severity [19, 21, 24, 25, 28, 29, 32, 33, 39, 40, 43, 48, 49]. Analyses adjusting for major confounders were reported in twelve studies [21–23, 25, 26, 28, 29, 31, 40, 47–49] and seven studies reported follow-up until all patients were either deceased or discharged [19, 20, 29, 31, 39, 46, 49]. Twelve studies contained more than 10% missing data [20, 21, 23–25, 30–32, 41, 43, 46, 49]. Twelve studies reported confidence precision estimate of association of ACEI/ARB with outcomes [21–29, 40, 47, 49]. One study included 17 people prescribed ACEI/ARB in the control group [40]. The overall strength of association between ACEI/ARB and outcomes was low (Table 3). None of the studies investigated the relationships between ACEI/ARB dose and outcome and only five studies reported whether ACEI/ARBs were continued during admission [19, 27, 40, 41, 46]. Only one study reported the association of continuation or discontinuation of medication during hospitalisation and outcome [27]. Biological plausibility and specificity of exposure and outcome were poorly evaluated in most studies and there was a high risk of confounding.

**Table 2:**
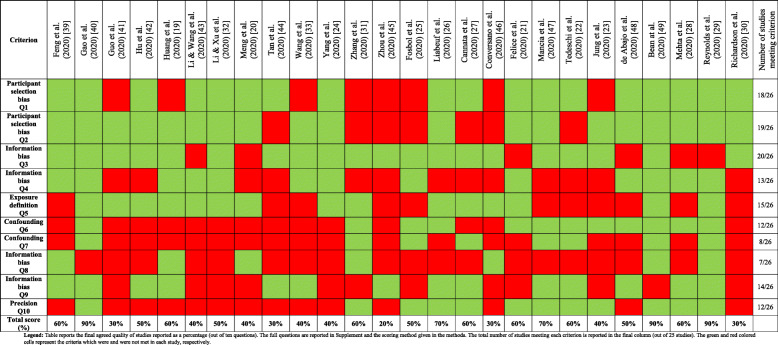
Quality of included studies based on standardised criterion

Legend: Table reports the final agreed quality of studies reported as a percentage (out of ten questions). The full questions are reported in Supplement and the scoring method given in “Material and methods.” The total number of studies meeting each criterion is reported in the final column (out of 25 studies). The green and red coloured cells represent the criteria which were and were not met in each study, respectively

**Table 3:**
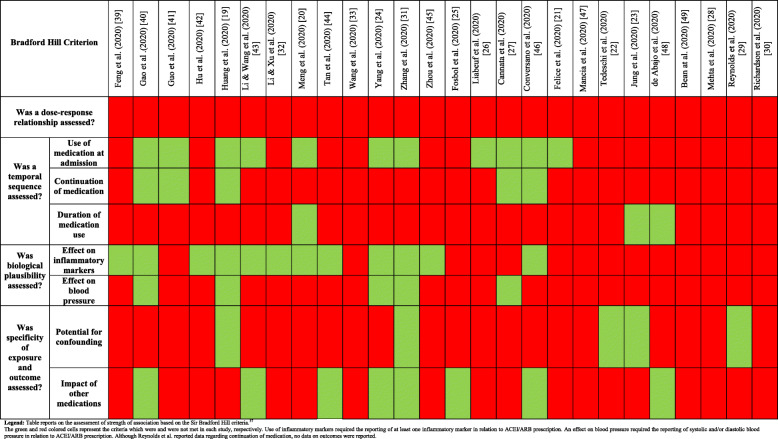
Strength of association between angiotensin converting enzyme inhibitor or angiotensin receptor blocker prescription and COVID-19 outcome based on standardised criteria

Legend: Table reports on the assessment of strength of association based on the Sir Bradford Hill criteria.^37^ The green and red coloured cells represent the criteria which were and were not met in each study, respectively. Use of inflammatory markers required the reporting of at least one inflammatory marker in relation to ACEI/ARB prescription. An effect on blood pressure required the reporting of systolic and/or diastolic blood pressure in relation to ACEI/ARB prescription. Although Reynolds et al. reported data regarding continuation of medication, no data on outcomes were reported

### Association of ACEI/ARB prescription with severe or critical disease outcome

**Fig. 2:**
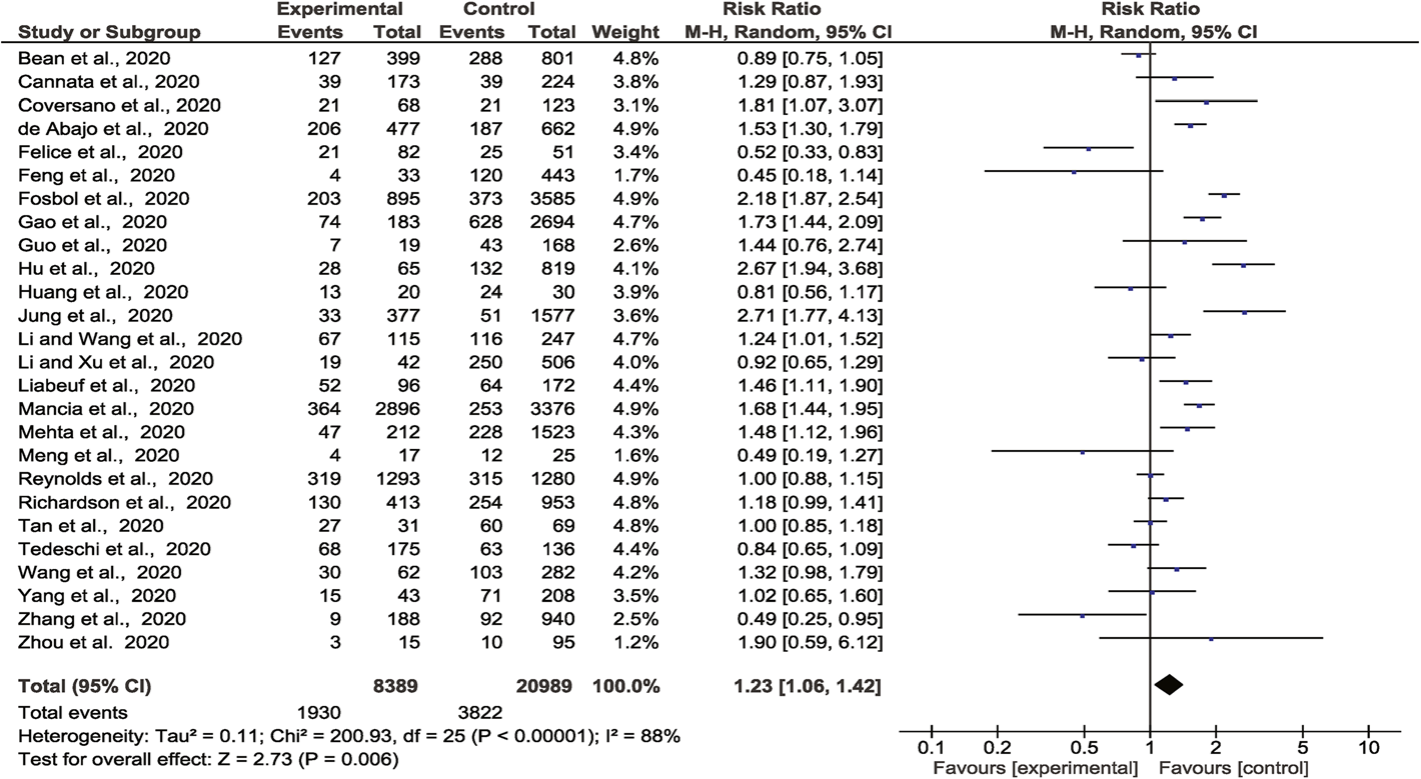
Forest plot of COVID-19 severity and association with prescription of ACEI/ARB

The meta-analysis incorporated 1930 severe or critical disease outcomes in 8389 people prescribed ACEI/ARB vs. 3822 severe or critical disease outcomes in 20,989 people not prescribed these medications from all 26 studies. The risk of severe or critical disease outcome was greater in people prescribed ACEI or ARB than those who were not (RR 1.23, 95% CI 1.06, 1.42, *Z* = 2.73, *p* = 0.006). There was a high degree of heterogeneity (*I*^2^ = 88%) (Fig. 2). Sub-group analyses found that people prescribed ACEI (RR 1.33, 95% CI 1.08, 1.63, *Z* = 2.68, *p* = 0.007) or ARB (RR 1.28, 95% CI 1.07, 1.52, *Z* = 2.68, *p* = 0.007) were at higher risk of severe or critical disease outcomes than those who were not prescribed these drugs (**S2****: Supp Fig 1-2**). Amongst people with a history of hypertension, there was no association between prescription of these medications and severe or critical disease outcomes (**S2****: Supp Fig 3**). Sensitivity analyses showed that when the analyses were restricted to people recruited from individual continents or high-quality studies, there was no significant association between ACEI/ARB prescription (or ACEI alone or ARB alone) and severe or critical disease outcomes (**S1****: Supp Table 9-11**). The funnel plots were asymmetrical (**S2****: Supp Fig 4-7)**.

### Association of ACEI/ARB prescription with COVID-19-related mortality

**Fig. 3:**
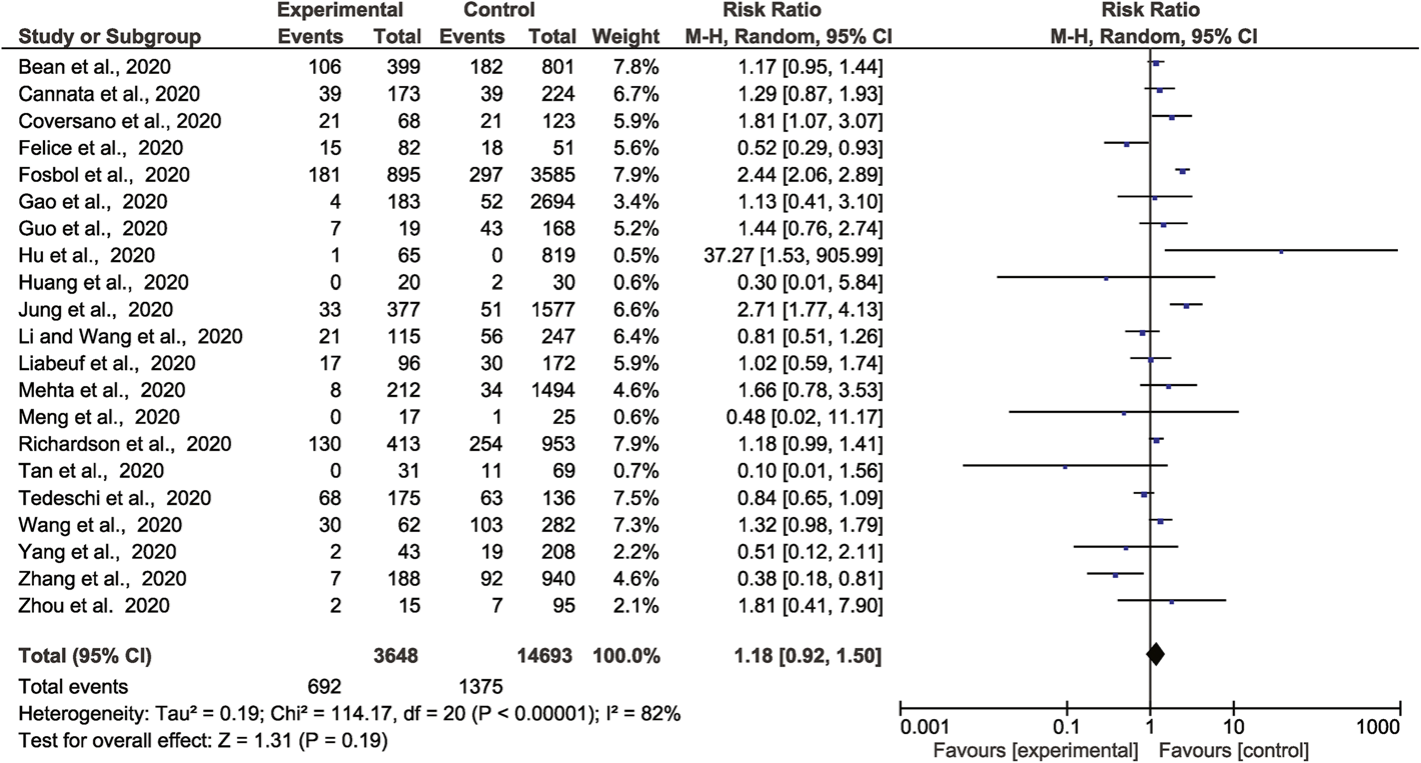
Forest plot of COVID-19 mortality and association with prescription of ACEI/ARB

**Fig. 4:**
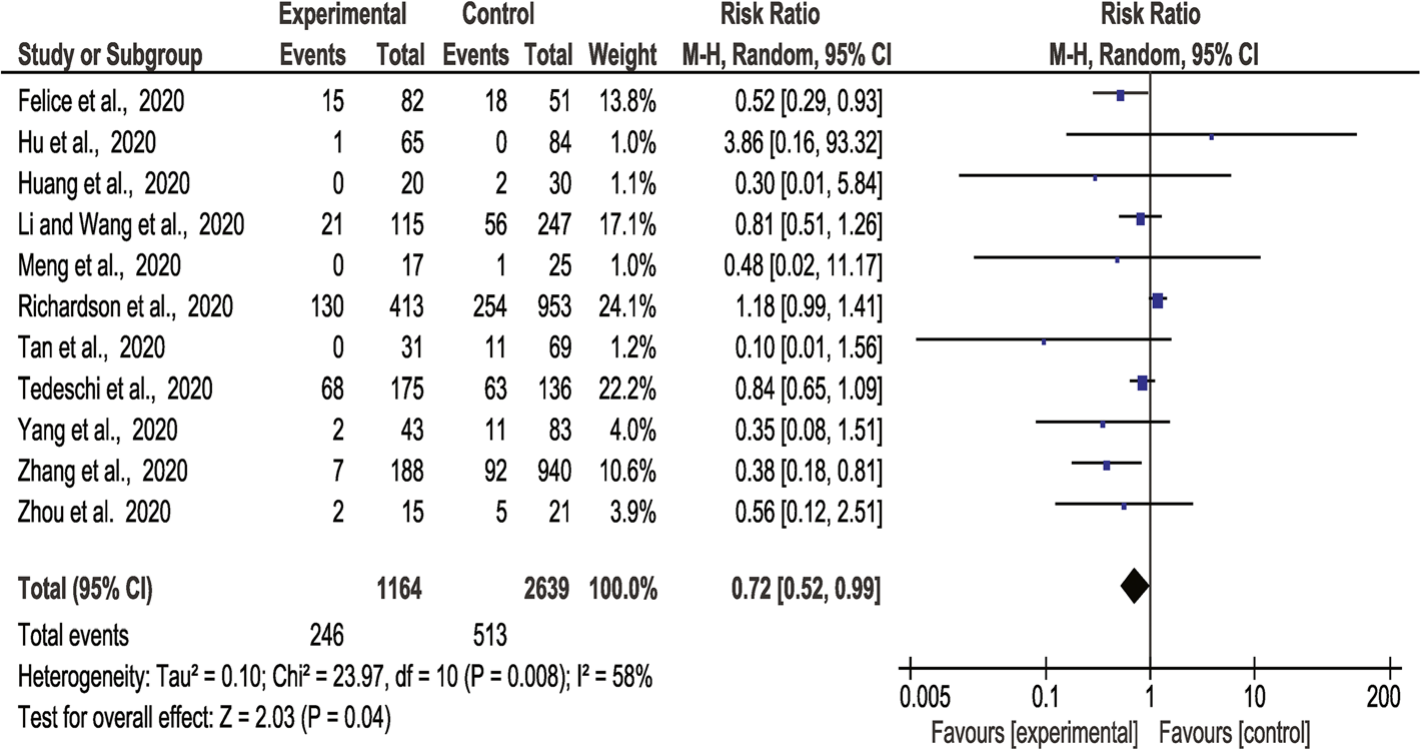
Forest plots of COVID-19 mortality and association with prescription of ACEI/ARB in people with a previous history of hypertension

There were 692 deaths in 3648 people prescribed ACEI/ARB vs. 1375 deaths in 14,693 people not prescribed these medications reported from 21 studies. Meta-analysis showed no statistically significant association between ACEI/ARB prescription and mortality (RR 1.18, 95% CI 0.92, 1.50, *Z* = 1.31, *p* = 0.19). There was a high degree of heterogeneity (*I*^2^ = 82%) (Fig. 3). Sub-group analyses found no significant associations between prescription of ACEI or ARB alone and mortality (**S2****: Supp Fig 8-9**). A sensitivity analysis focused on people with a history of hypertension alone incorporated 246 deaths in 1164 people prescribed ACEI/ARB vs. 513 deaths in 2639 people not prescribed these medications from 11 studies. This found a lower risk of death in people prescribed ACEI or ARB (RR 0.72, 95% CI 0.52, 0.99, *Z* = 2.03, *p* = 0.04) with a moderate degree of heterogeneity (*I*^2^ = 58%) (Fig. 4). Sensitivity analyses suggested that findings for the main analysis and class-specific analyses were not dependent on the inclusion of any individual studies and were similar when restricted to people recruited from individual continents or high-quality studies (**S1****: Supp Tables 11-13**). The finding of the analysis restricted to people with hypertension was largely dependent on the inclusion of two studies (**S1****: Supp Table 13)**. The funnel plots were asymmetrical (**S2****: Supp Fig 4-7)**.

## Discussion

Ideally, choices about drug prescription should be based on data from randomised controlled trials; however, such studies are not easy to perform during a pandemic. To our knowledge, this is one of the largest and only meta-analysis to pool data exclusively from published observational studies to examine whether an association exists between ACEI or ARB prescription and COVID-19 outcome. The main meta-analyses showed that people prescribed ACEI/ARB had a higher risk of severe or critical disease outcomes. Observational studies are subject to biases that must be considered during interpretation. It is therefore important to highlight that people prescribed ACEI/ARB were older and more likely to be male and had higher prevalence of a number of co-morbidities, including hypertension, diabetes, IHD, CCF, CKD and COPD, that have been associated with worse outcome from COVID-19 [50–53]. Furthermore, the association between ACEI/ARB and severe or critical disease outcomes was not robust, being lost in sensitivity analyses restricted to people with hypertension, those recruited from individual continents or high-quality studies. There was also no association between drug prescription and mortality in most of the analyses except the one restricted to people with hypertension in which there was a 28% lower mortality in people prescribed either ACEIs or ARBs. The latter finding was not however robust in sensitivity analyses. Overall, these findings suggest that there is no robust published observational data that ACEI/ARB prescription is associated with worse or better outcome from COVID-19. Thus, the findings suggest no evidence to stop or start these medications in people admitted to hospital with COVID-19.

Since our search was conducted, there have been other meta-analyses published examining the relationship between ACEI/ARB prescription and COVID-19 outcomes [54–62]. These analyses (amongst others not described here) differ widely in their total number of included studies, study type(s) included, patient populations, inclusion of retracted studies [56], undertaking of quality assessment and use of quality assessment outcomes in interpreting the results and inclusion of pre-print articles [56, 59–62]. These differences likely contributed to the conflicting findings between this and similar studies. Many analyses have found no significant difference in either COVID-19 severity or mortality related to ACEI/ARB prescription [55, 56, 58, 61] whilst others have found significant reductions either for the whole population [54, 57, 62] or in sub-analyses, such as for patients with hypertension [59, 60]. No studies have found an increased risk of severe disease or mortality from COVID-19 in relation to ACEI/ARB prescription. Some analyses also examined the association between ACEI/ARB prescription and the risk of COVID-19 infection, which also varied depending on the class of agent (significant reduction with ACEI but not ARB) [57], and sub-analyses of the patient population (significant increase but not when adjusting for patients with hypertension) [58].

The outcomes from COVID-19 found in this study were likely influenced by treatments received whilst in the hospital including the use of systemic steroids and anti-viral medication, but these were poorly reported in the included studies. At the time of writing, dexamethasone is the only medication with a proven reported mortality benefit in people infected with COVID-19 based on high-quality randomised controlled trial data [63]. Dexamethasone reduced deaths by one-third in patients receiving invasive mechanical ventilation compared to standard care [63]. Very few studies included in the current meta-analysis reported the in-patient treatment differences between people prescribed and not-prescribed ACEI/ARB, and in the studies that did report in-patient treatments, several studies reported differences in the use of supportive therapies including greater use of corticosteroids during hospital admission in people prescribed ACEI/ARB [20, 23, 39, 41, 44].

Key elements to any systematic review are evaluations of study quality and strength of association. Only three (12%) of the included studies were deemed high quality. The strength of association between exposure and outcome in the studies was also limited. Additionally, given the retrospective design of most studies, selection bias remains an important problem [31]. Although not explored in the current meta-analysis, whether the prescription of ACEI/ARB has an association with the incidence of COVID-19 still remains unclear although a lack of such association has been reported [64], though others are conflicting [57, 58].

This meta-analysis had a number of strengths and limitations. Strengths included the systematic approach and careful examination of the quality and strength of association using standardised tools and extensive sensitivity analyses [13, 35, 65]. It is possible that dual reporting of data from similar populations may have occurred. Sensitivity analyses were performed to minimise the effect of this on the overall outcome. Adjustment of analyses for confounding factors, such as older age, hypertension, diabetes, IHD and CKD, was not possible, and it is likely that the association of ACEI/ARB prescription and severe or critical disease outcomes resulted from residual confounding due to disproportional prevalence of risk factors in the two groups. We only included studies published in English in the search period listed. Lastly, the impact of protopathic and residual bias due to cessation or initiation of ACEI/ARB during hospital admission was not adequately assessed in almost all the included studies and is an important consideration.

## Conclusions

This meta-analysis of published observational studies suggests that there is no robust published observational data that ACEI/ARB prescription is associated with worse or better outcome from COVID-19. Thus, similar to the recommendations from other studies, the findings suggest no evidence to stop or start these drugs in people admitted to the hospital with COVID-19.

## Supplementary Information

**Additional file 1: S1:** Additional Tables and Data.

**Additional file 2: S2:** Additional Plots and Figures.

## Data Availability

Data are available from the corresponding author upon reasonable request.

## Abbreviations

ACE2: Angiotensin-converting enzyme 2
ACEI: Angiotensin-converting enzyme inhibitor
ARB: Angiotensin receptor blocker
CCF: Congestive cardiac failure
CI: Confidence interval
CKD: Chronic kidney disease
COPD: Chronic obstructive pulmonary disease
CRP: C-reactive protein
ICD-10: International Statistical Classification of Diseases and Related Health Problems Version 10
IHD: Ischaemic heart disease
MOOSE: Meta-analysis Of Observational Studies in Epidemiology
PRISMA: Preferred Reporting Items for Systematic Reviews and Meta-Analyses
RR: Risk ratio
WHO: World Health Organization
