# Supplementary material for: Meta-analysis of the association between angiotensin pathway inhibitors and COVID-19 severity and mortality
Source: Syst Rev. 2021 Sep 7;10:243. doi: 10.1186/s13643-021-01802-6 (PMC8421238; doi:10.1186/s13643-021-01802-6)
Supplement: Supplementary file 1 — Additional file 1: S1: Additional Tables and Data. [file 13643_2021_1802_MOESM1_ESM.docx]

Key words: 2019 novel coronavirus disease, 2019 novel coronavirus infection, 2019-nCoV disease, 2019-nCoV infection, COVID19, COVID-19, coronavirus disease 2019 or coronavirus disease-19” in combination with MeSH terms and title words for hypertension and ACEI or ARB “hypertens*, antihypertens*, anti-hypertens*, angiotens*, A2RB, ARB, ACE, ACEI, Kininase*

Search string: (((((((((2019 novel coronavirus disease[Title]) OR 2019 novel coronavirus infection[Title]) OR 2019-nCoV disease[Title]) OR 2019-nCoV infection[Title]) OR COVID19[Title]) OR COVID-19[Title]) OR coronavirus disease 2019[Title]) OR coronavirus disease-19[Title]) AND ((((((((((hypertens*[Title]) OR antihypertens*) OR anti-hypertens*) OR angiotens*) OR A2RB*) OR ARB*) OR ACE*) OR ACEI*) OR Kinin*) OR Inhibit*))

**S1: Additional Tables and Data**

**Supp Text Box 1: Text box showing complete search strategy**

**Supp Table 1:** **Definitions and reporting within included studies**

| **Author** | **Method of COVID-19 diagnosis** | **Definition of hypertension** | **Definition of antihypertensive use** | **Antihypertensive dose** | **Was antihypertensive used during admission** | **Definition of mortality and cause of death** | **Definition of disease severity** | **Inflammatory markers reported?** | **In-patient treatment differences** |
| --- | --- | --- | --- | --- | --- | --- | --- | --- | --- |
| Feng et al.  (2020) ^(1)^ | Met WHO criteria | Not reported | Electronic medical records | Not reported | Not reported | In hospital death | Guideline based | Yes | Antivirals, antibiotics, antifungals, corticosteroids for differing severities. |
| Gao et al.  (2020) ^(2)^ | Met WHO criteria | SBP/DBP above threshold or antihypertensive use | At admission | Not reported | Yes | In hospital death | Guideline based | Yes | Not reported |
| Guo et al.  (2020) ^(3)^ | Met WHO criteria | Medical records | At admission | Not reported | Not reported | In hospital death | Not reported | Yes | Possible differences in supportive treatment between groups |
| Hu et al.  (2020) ^(4)^ | Met WHO criteria | Not reported | Not reported | Not reported | Not reported | In hospital death | ICU admission | Yes | No differences in supportive treatment between groups |
| Huang et al.  (2020) ^(5)^ | Met WHO criteria | Self-reported | Self-reported | Not reported | Yes | In hospital death | Guideline based | Yes | No differences in supportive treatment between groups |
| Li & Wang et al.  (2020) ^(6)^ | Met WHO criteria | SBP/DBP above threshold or antihypertensive use | At admission + continued during admission | Not reported | Yes | Not reported | Guideline based | Yes | Not reported |
| Li & Xu et al.  (2020) ^(7)^ | Met WHO criteria | ICD-10 based | At admission | Not reported | Not reported | In hospital death | American Thoracic Soc. pneumonia guideline or requiring ventilator support | Yes | Antivirals given to severe group |
| Meng et al.  (2020) ^(8)^ | Met WHO criteria | No clear definition given | Not reported | Not reported | Not reported | Guideline based | Based on standardised criteria | Yes | Steroids, antivirals, antibiotics, IVIG. More steroids and IVIG in ACE/ARB |
| Tan et al.  (2020) ^(9)^ | Not reported | Not reported | Not reported | Not reported | Not reported | In hospital death | Unclear | Yes | Differences in supportive treatment (systemic steroids, BB) between groups |
| Wang et al.  (2020) ^(10)^ | Met WHO criteria | Medical records | Electronic medical records | Not reported | Not reported | 28-day mortality | Guideline based | Yes | Not reported |
| Yang et al.  (2020) ^(11)^ | Met WHO criteria | No clear definition given | Prior to admission | Not reported | Not reported | Not reported | Based on standardised criteria | No | Not reported |
| Zhang et al.  (2020) ^(12)^ | Met WHO criteria + Some CT-only diagnoses | Guideline based definition | Clinical record | Not reported | Yes, but not in all participants | 28-day all-cause death/ ARDS/ Shock | Not reported, invasive ventilation used as a surrogate | Yes | Traditional Chinese medicine, 88.8% antivirals/statin higher in ACEI/ARB |
| Zhou et al.  (2020) ^(13)^ | Chinese-based guidelines | Medical records | Electronic medical records | Not reported | Not reported | In hospital death | Transfer to high level hospital used as a surrogate | Yes | Not reported |
| Fosbol et al.  (2020) ^(14)^ | Met WHO criteria, some not PCR confirmed | ICD-8 based | Electronic medical records | Not reported | Not reported | In hospital death | ICU admission and ICD code | No | Not reported |
| Liabeuf et al.  (2020) ^(15)^ | Met WHO criteria | Medical records | Home drug prescription | Not reported | Not reported | Death before ICU admission | ICU admission | No | Not clearly stated |
| Cannata et al.  (2020) ^(16)^ | Not reported | Not reported | At admission | Not reported | Yes | In hospital death | Not reported | No | Not reported |
| Conversano et al.  (2020) ^(17)^ | Met WHO criteria | Not reported | At admission | Not reported | Yes, but not in all participants | In hospital death | Not reported | Yes | Not reported but survivors had longer ICU admission duration |
| Felice et al.  (2020) ^(18)^ | Met WHO criteria | Any patient on an antihypertensive drug | Not reported | Not reported | Not reported | In hospital death | ICU admission | No | Not reported |
| Mancia et al.  (2020) ^(19)^ | Met WHO criteria | ICD-9 based | 2019 prescription | Not reported | Not reported | Critical or fatal infection composite | | No | Not clearly stated |
| Tedeschi et al.  (2020) ^(20)^ | Not reported | No clear definition given | Not reported | Not reported | Not reported | In hospital death | Not reported | No | Not reported |
| Jung et al.  (2020) ^(21)^ | Met WHO criteria | KCD-7 (ICD-10 modified) | Use 30 days prior admission | Collected but not analysed | Not reported | In hospital death | Mechanical ventilation used as surrogate | No | Supportive treatments more common in ACEI/ARB group |
| de Abajo et al.  (2020) ^(22)^ | Met WHO criteria | Not reported | Electronic medical records | Not reported | Not reported | In hospital death | Composite of death, ICU admission | No | Not reported |
| Bean at al.  (2020) ^(23)^ | Met WHO criteria | Guideline based definition | Prescription based | Reported in supplement | Up to a year prior to admission | In-hospital death within 21 days | Composite of death, ICU admission within 21 days | Yes | Not reported |
| Mehta et al.  (2020) ^(24)^ | Met WHO criteria | Medical records | Electronic medical records | Not reported | Not reported | Not reported | ICU admission used as a surrogate | No | Not reported |
| Reynolds et al.  (2020) ^(25)^ | Met WHO criteria | ICD-10 based | Electronic record 1.5 years prior to testing | Not reported | Record use a month prior to admission | Not reported | Composite of death, ICU admission, mechanical ventilation | No | Not reported |
| Richardson et al.  (2020) ^(26)^ | Met WHO criteria | No clear definition given | Electronic medical records | Not reported | 50% continued at admission, 107 newly commenced | In hospital death | Not given, used ICU admission as a surrogate | Yes | In patients who died people on ACEI/ARB needed ICU/mechanical ventilation |

**Legend:** SBP= systolic blood pressure, DBP= diastolic blood pressure, ICD-10= International Classification of Diseases, 10th Revision, WHO= World Health Organization, ATS= American Thoracic Society, ACEI= Angiotensin enzyme inhibitor, ACEI= angiotensin-converting enzyme inhibitor, ARB= angiotensin-2 receptor blocker, HTN= hypertension, DM= diabetes mellitus, IHD= ischemic heart disease/coronary artery disease, CCF= congestive cardiac failure. CT= Computed tomography. ICU= intensive care unit.

**Supp Table 2: Summary description of includes studies, including patient comorbidities and anti-hypertensive agents used**

| **Study** | **Total sample** | **Age** | **Male Sex** | **Smoker** | **DM** | **COPD** | **CKD** | **IHD** | **CCF** | **HTN** | **Age in**  **HTN** | **Male Sex in HTN** | **ACEI / ARB** | **ACEI** | **ARB** | **CCB** | **Diuretic** | **BB** | **AB** |
| --- | --- | --- | --- | --- | --- | --- | --- | --- | --- | --- | --- | --- | --- | --- | --- | --- | --- | --- | --- |
| Feng et al.  (2020) ^(1)^ | 476 | 53  (40-64) | 271  (57%) | 44  (10%) | 49  (10%) | 22  (5%) | 4  (1%) | 38  (8%) | | 113  (24%) | NR | NR | 33  (7%) | 8  (2%) | 27  (6%) | 62  (13%) | | | |
| Gao et al.  (2020) ^(2)^ | 2877 | NR | 1470  (51%) | 190  (7%) | 387  (13%) | 31  (1%) | 29  (1%) | 221  (8%) | 23  (1%) | 850  (30%) | 64  (SD 11) | 443  (52%) | 200  (6%) | 58  (2%) | 142  (5%) | 610  (21%) | 54  (2%) | 161  (6%) | NR |
| Guo et al.  (2020) ^(3)^ | 187 | 59  (SD 15) | 91  (49%) | 18  (10%) | 28  (15%) | 4  (2%) | 6  (3%) | 21  (11%) | 8  (4%) | 61  (33%) | NR | NR | 19  (10%) | NR | NR | NR | NR | NR | NR |
| Hu et al.  (2020) ^(4)^ | 884 | NR | 455  (51%) | NR | 65  (7%) | 5  (1%) | 8  (1%) | 15  (2%) | NR | 149  (17%) | 57  (50-66) | 88  (59%) | 65  (7%) | NR | NR | 84  (10%) | | | |
| Huang et al.  (2020) ^(5)^ | 50 | NR | 27  (54%) | NR | 4  (8%) | 1  (2%) | NR | 1  (2%) | NR | 50  (100%) | 62  (SD 13) | 27  (54%) | 20  (40%) | NR | NR | 36  (72%) | 3  (6%) | 11  (22%) | NR |
| Li & Wang et al.  (2020) ^(6)^ | 362^$^ /  1178 | 56  (38-67) | 545  (46%) | NR | 203  (17%) | 54  (5%) | 44  (4%) | 103  (9%) | 21  (2%) | 362  (31%) | 66  (59-73) | 189  (52%) | 115  (10%) | 35  (3%) | 83  (7%) | 250  (21%) | NR | 14  (4%) | NR |
| Li & Xu et al.  (2020) ^(7)^ | 548 | 60  (48-69) | 279  (51%) | 41  (8%) | 83  (15%) | 17  (3%) | 10  (2%) | 34  (6%) | NR | 166  (30%) | NR | NR | 42  (8%) | NR | NR | NR | NR | NR | NR |
| Meng et al.  (2020) ^(8)^ | 42^$^ /  417 | 65  (56–69) | 24  (57%) | NR | 13  (31%) | NR | NR | 13  (31%) | NR | 42/51  (100%) | 65  (56-69) | 24  (5%) | 17  (41%) | 2  (5%) | 15  (36%) | 28  (67%) | 4  (10%) | 10  (24%) | NR |
| Tan et al.  (2020) ^(9)^ | 100^$^ /  204 | NR | 51  (51%) | NR | 28  (28%) | 9  (9%) | 9  (9%) | 18  (18%) | NR | 100  (100%) | NR | 51  (51%) | 31  (31%) | 4  (4%) | 27  (27%) | 39  (39%) | NR | 13  (13%) | 2  (2%) |
| Wang et al.  (2020) ^(10)^ | 344 | 64  (57-72) | 179  (52%) | NR | 64  (19%) | 16  (5%) | NR | 40  (12%) | NR | 141 (41%) | NR | NR | 62  (18%) | 62  (18%) | NR | NR | NR | NR | NR |
| Yang et al.  (2020) ^(11)^ | 251^$^ /  462 | NR | 123  (49) | NR | 55  (22%) | 12  (5%) | 4  (2%) | 35  (14%) | | 126  (54%) | 66  (61-73) | 62  (49%) | 43  (17%) | NR | NR | NR | NR | NR | NR |
| Zhang et al.  (2020) ^(12)^ | 3430 | 57  (45-65) | 1675  (49%) | NR | 388  (11%) | 19  (1%) | 52  (2%) | 178  (5%) | NR | 1128  (33%) | 64  (56-69) | 603  (54%) | 188  (6%) | 31  (1%) | 157  (5%) | 592  (17%) | 300  (9%) | 334  (10%) | 35  (1%) |
| Zhou et al.  (2020) ^(13)^ | 110 | 58  (SD 14) | 60  (55%) | NR | 11 (10%) | 3  (3%) | 2  (2%) | 10  (9%) | NR | 36  (33%) | 65  (SD 10) | 19  (53%) | 15  (14%) | NR | NR | 21  (19%) | | | |
| Fosbol et al.  (2020) ^(14)^ | 4480 | 55  (41-72) | 2144  (48%) | NR | 411  (9%) | 634  (14%) | 172  (4%) | 379  (8%) | 243  (5%) | 843  (19%) | NR | NR | 895  (20%) | 377  (8%) | 530  (12%) | 487  (11%) | 368  (8%) | 525  (12%) | NR |
| Liabeuf et al.  (2020) ^(15)^ | 268 | 73  (61-84) | 155  (58%) | 9  (3%) | 55  (21%) | 26  (10%) | 19  (7%) | 33  (12%) | 30  (11%) | 152  (57%) | NR | NR | 96  (36%) | NR | NR | 57  (21%) | 58  (22%) | 73  (27%) | NR |
| Cannata et al.  (2020) ^(16)^ | 397 | NR | NR | NR | NR | NR | NR | NR | NR | NR | NR | NR | 173  (44%) | NR | NR | NR | NR | NR | NR |
| Conversano et al.  (2020) ^(17)^ | 191 | 63  (SD 15) | 131  (69%) | NR | 28  (15%) | 10  (5%) | 50  (26%) | 28  (15%) | 9  (5%) | 96  50% | 71  (SD 11) | 73  (76%) | 69  (36%) | 35  (18%) | 33  (17%) | 25  (13%) | 16  (8%) | 50  (26%) | NR |
| Felice et al.  (2020) ^(18)^ | 133 | 73  (SD 13) | 86  (65%) | 3  (2%) | 34  (25%) | 14  (11%) | NR | 56  (42%) | 24  (18%) | 133  (100%) | 72  (62-82) | 86  (65%) | 82  (62%) | 40  (31%) | 42  (32%) | 51  (38%) | | | |
| Mancia et al.  (2020) ^(19)^ | 6272 | 68  (SD 13) | 3969  (63%) | NR | NR | 188  (3%) | 181  (3%) | 473  (8%) | 323  (5%) | NR | NR | NR | 2896  (46%) | 1502  (24%) | 1394  (22%) | 1446  (23%) | 1902  (30%) | 1826  (29%) | NR |
| Tedeschi et al.  (2020) ^(20)^ | 311^$^ /  609 | 68  (55-80) | 410  (68%) | 106  (17%) | 100  (16%) | 68  (11%) | NR | 165  (27%) | NR | 311  (51%) | 76  (67-83) | 225  (72%) | 175  (29%) | 99  (32%) | 76  (25%) | 76  (25%) | | | |
| Jung et al.  (2020) ^(21)^ | 1954^$^ / 5179 | NR | NR | NR | NR | NR | NR | NR | NR | 542  (28%) | NR | NR | 377  (19%) | 20  (1%) | 365  (19%) | NR | NR | NR | NR |
| de Abajo et al.  (2020) ^(22)^ | 1139 | 69  (SD 15) | 695  (61%) | NR | 310  (27%) | 119  (10%) | 89  (8%) | 119  (10%) | 80  (7%) | 617  (54%) | NR | NR | 477  (42%) | 240  (21%) | 237  (21%) | 212  (19%) | 347  (31%) | 200  (18%) | 40  (4%) |
| Bean at al.  (2020) ^(23)^ | 1200 | 68  (SD 17) | 686  (57%) | NR | 418 (35%) | 121 (10%) | 206 (17%) | 160 (13%) | 107 (9%) | 645 (54%) | NR | NR | 399  (33%) | 260  (22%) | 147  (12%) | NR | NR | 337  (28%) | NR |
| Mehta et al.  (2020) ^(24)^ | 1735 | NR | 955  (55%) | NR | NR | NR | NR | NR | NR | NR | NR | NR | 212  (12%) | 116  (7%) | 98  (6%) | NR | NR | NR | NR |
| Reynolds et al.  (2020) ^(25)^ | 5984 | NR | NR | NR | NR | NR | NR | NR | NR | 2573  (43%) | NR | NR | NR | NR | NR | NR | NR | NR | NR |
| Richardson et al.  (2020) ^(26)^ | 1366^$^ / 5700 | 63  (52-75) | 3437  (60%) | 558  (16%) | 1808  (32%) | 287  (5%) | 268  (5%) | 595  (10%) | 371  (7%) | 3026  (53%) | NR | NR | 413  (30%) | 168  (12%) | 245  (18%) | NR | NR | NR | NR |

**Legend:** Data are presented as number/total (percentage), median (interquartile range) or where specified mean (± Standard deviation SD). Age was rounded to nearest integer. NR= not reported. DM= diabetes mellitus, COPD= chronic obstructive pulmonary disease, CKD= chronic kidney disease, IHD= ischemic heart disease, CCF= congestive cardiac failure, HTN= hypertension, ACEI= angiotensin-converting enzyme inhibitor, ARB= angiotensin-2 receptor blocker, CCB= calcium channel blocker, BB= beta-adrenergic blocker, AB= alpha blocker.

^ Reported vascular disease which was inferred to be coronary artery disease, # Reported both matched and unmatched data in cases vs. controls. Matched data reported for comparison, $ Clinical data was available for only 251 of 462 patients in Yang et al, for 362 of 1178 for Li and Wang, for 42 of 417 patients in Meng et al, for 1954 hospitalized patients out of 5179 for Jung et al, 1366 of 5700 patients in Richardson et al, 100 of 204 for Tan et al, and for Reynolds et al include 5984 people with COVID-19, data were reported for 4357 people with hypertension. Summary stats presented are for the entire cohort, and medication use stats for the clinical data sub-cohort. In Meng et al. nine patients (17.6%) with Grade 1 hypertension did not take any antihypertensive drugs during hospitalization and were excluded from the subsequent analysis. Yang et al. reported combined data for IHD or CCF. Richardson et al. only had clinical data for 46.2% of admitted patients and medication data was only available for (92%) of the included patients. For Li et.al. we assumed all those with hypertension would be taking medications. In Richardson et al. 267 people were prescribed an ACEI at home and 189 people were prescribed an ARB. Richardson et al. smoking data may include ex-smokers. $ Clinical data was available for only 251 of 462 patients in Yang et al, for 362 of 1178 for Li and Wang, for 42 of 417 patients in Meng et al, for 1954 hospitalized patients out of 5179 for Jung et al, 1366 of 5700 patients in Richardson et al, 100 of 204 for Tan et al, and for Reynolds et al include 5984 people with COVID-19, data were reported for 4357 people with hypertension. In Meng et al. nine patients (17.6%) with Grade 1 hypertension did not take any antihypertensive drugs during hospitalization and were excluded from the subsequent analysis. Yang et al. reported combined data for IHD or CCF. Richardson et al. only had clinical data for 46.2% of admitted patients and medication data was only available for (92%) of the included patients. For Li et.al. we assumed all those with hypertension would be taking medications. In Richardson et al. 267 people were prescribed an ACEI at home and 189 people were prescribed an ARB. Richardson et al. smoking data may include ex-smokers.

**Supp Table 3: Blood pressure, laboratory results, and severe or critical disease outcome and mortality outcomes for each study**

|  | **Characteristics of people prescribed ARBs/ACEI (n=8389)** | | | | | | | | **Characteristics of people not prescribed ARBs/ACEI (n=20989)** | | | | | | |
| --- | --- | --- | --- | --- | --- | --- | --- | --- | --- | --- | --- | --- | --- | --- | --- |
| **Study** | **People using**  **ACEI / ARB classes** | **Systolic blood**  **pressure**  **(mm Hg)** | **Diastolic blood**  **pressure**  **(mm Hg)** | **C-reactive protein**  **(mg/L)** | **Severe or critical disease outcome** | **Use of Systemic Steroids** | **Use of Anti-viral medication** | **Mortality** | **Systolic blood**  **pressure**  **(mm Hg)** | **Diastolic blood**  **pressure**  **(mm Hg)** | **C-reactive protein**  **(mg/L)** | **Severe or critical disease outcome** | **Mortality** | **Use of Systemic Steroids** | **Use of Anti-viral medication** |
| Feng et al.  (2020) ^(1)^ | 8 / 27 | NR | NR | NR | 4 | NR | NR | NR | NR | NR | NR | 120 | NR | NR | NR |
| Gao et al.  (2020) ^(2)^ | 58 / 142 | 136  (125-152) | 84  (76-92) | 2.7  (1.2-11.7) | 74 | NR | NR | 4 | NR | NR | NR | 628 | 52 | NR | NR |
| Guo et al.  (2020) ^(3)^ | NR | NR | NR | NR | 7 | NR | NR | 7 | NR | NR | NR | 43 | 43 | NR | NR |
| Hu et al.  (2020) ^(4)^ | NR | NR | NR | 21  (7-41) | 28 | 19 (29%) | NR | 1 | NR | NR | NR | 132 | 0 | 28 (33%) | NR |
| Huang et al.  (2020) ^(5)^ | NR | 125  (SD 17) | 77  (SD 12) | 81  (14-178) | 13 | 10 (50%) | 20 (100%) | 0 | 129  (SD 20) | 78  (SD 12) | 49  (7-125) | 24 | 2 | 14 (47%) | 29 (97%) |
| Li & Wang et al.  (2020) ^(6)^ | 35 / 83 | NR | NR | 21  (3-52) | 67 | NR | NR | 21 | NR | NR | 26  (4-60) | 116 | 56 | NR | NR |
| Li & Xu et al.  (2020) ^(7)^ | NR | NR | NR | NR | 19 | NR | NR | NR | NR | NR | NR | 250 | NR | NR | NR |
| Meng et al.  (2020) ^(8)^ | 2 / 15 | NR | NR | NR | 4 | NR | NR | 0 | NR | NR | NR | 12 | 1 | NR | NR |
| Tan et al.  (2020) ^(9)^ | 4 / 27 | NR | NR | 24  (4-43) | 27 | 3 (10%) | 19 (61%) | 0 | NR | NR | 24  (7-63) | 60 | 11 | 26 (38%) | 52 (75%) |
| Wang et al.  (2020) ^(10)^ | 62 / 0 | NR | NR | NR | 30 | NR | NR | 30 | NR | NR | NR | 103 | 103 | NR | NR |
| Yang et al.  (2020) ^(11)^ | NR | 129  (120-140) | 77  (70-85) | 11.5  (4.0-58) | 15 | 16  (37%) | 30  (70%) | 2 | NR | NR | NR | 71 | 19 | 58  (28%) | 166  (80%) |
| Zhang et al.  (2020) ^(12)^ | 31 / 157 | 133  (123-146) | 80  (72-88) | >5  (59%) | 9^ | NR | NR | 7 | 134  (121-145) | 80  (73-86) | >5  (69%) | 92 | 92 | NR | NR |
| Zhou et al.  (2020) ^(13)^ | NR | NR | NR | NR | 3 | NR | NR | 2 | NR | NR | NR | 10 | 7 | NR | NR |
| Fosbol et al.  (2020) ^(14)^ | 377 / 530 | NR | NR | NR | 203 | NR | NR | 181 | NR | NR | NR | 373 | 297 | NR | NR |
| Liabeuf et al.  (2020) ^(15)^ | NR | NR | NR | NR | 52 | NR | NR | 17 | NR | NR | NR | 64 | 30 | NR | NR |
| Cannata et al.  (2020) ^(16)^ | NR | NR | NR | NR | 7 / 39 | NR | NR | 7 / 39 | NR | NR | NR | 39 | 39 | NR | NR |
| Conversano et al.  (2020) ^(17)^ | 35 / 33 | NR | NR | NR | 21 | NR | NR | 21 | NR | NR | NR | 21 | 21 | NR | NR |
| Felice et al.  (2020) ^(18)^ | 40 / 42 | 141  (SD 21) | 80  (SD 14) | 9.8  (SD 8.5) | 21 | NR | NR | 15 | 136  (SD 29) | 80  (SD 18) | 10.9  (SD 9.3) | 25 | 18 | NR | NR |
| Mancia et al.  (2020) ^(19)^ | 1502 / 1394 | NR | NR | NR | 364 | NR | NR | NR | NR | NR | NR | 253 | NR | NR | NR |
| Tedeschi et al.  (2020) ^(20)^ | 99 / 76 | NR | NR | NR | 68 | NR | NR | 68 | NR | NR | NR | 63 | 63 | NR | NR |
| Jung et al.  (2020) ^(21)^ | 20 / 365 | NR | NR | NR | 33 | 34 (9%) | 179 (47%) | 33 | NR | NR | NR | 51 | 51 | 41 (3%) | 603 (38%) |
| de Abajo et al.  (2020) ^(22)^ | 240 / 237 | NR | NR | NR | 206 | NR | NR | NR | NR | NR | NR | 187 | NR | NR | NR |
| Bean at al.  (2020) ^(23)^ | 260/147 | 126 (SD 28) | 71  (SD 18) | NR | 127 | NR | NR | 106 | 123  (SD 26) | 71  (SD 18) | NR | 288 | 182 | NR | NR |
| Mehta et al.  (2020) ^(24)^ | 116 / 98 | NR | NR | NR | 47 | NR | NR | 8/211 | NR | NR | NR | 228 | 34/1494 | NR | NR |
| Reynolds et al.  (2020) ^(25)^ | 584 / 739 | NR | NR | NR | 319 | NR | NR | NR | NR | NR | NR | 315 | NR | NR | NR |
| Richardson et al.  (2020) ^(26)^ | 168 / 245 | NR | NR | NR | 130 | NR | NR | 130 | NR | NR | NR | 254 | 254 | NR | NR |

**Legend:** Data are presented as number/total (percentage), median (interquartile range) or where specified mean (± Standard deviation SD) depending on how studies reported these values. ACEI= angiotensin-converting enzyme inhibitor, ARB= angiotensin-2 receptor blocker. SBP= systolic blood pressure, DBP= diastolic blood pressure, CRP= C-reactive protein. NR= not reported, ** only for hypertension group, *** only for ARB/ACEI group, # Reported both matched and unmatched data in cases vs. controls. Matched data reported for comparison, #Reported both matched and unmatched data in cases vs. controls. Matched data reported for comparison, ^Value was computed as was not directly reported in the study. For Richardson et al. Only ICU cases were utilised as severe cases so as to not count one participant more than once. In Li & Wang et al. three patients used ACEI + ARB at the same time, so the total number of ACEIs/ARBs was 3 less than the sum of the 2 drugs. X/Y numbers indicate the mortality reported in people with and without hypertension/ people with hypertension only. For Reynolds et al. out of 2573 patients with hypertension, 634 (24.6%) had severe COVID-19 outcomes, 422 had ICU admission, 165 mechanical ventilation, and death in 343 until 15th April 2020. In Li and Wang et al., outcomes were only reported for people with hypertension and as the use of ACEI/ARB could not be excluded in people without hypertension only this data was used in the main comparison. In Richardson et al. 140/168 people continued to take ACEI during hospitalisation, and 194/245 people continued to take ARB. Only data on those who died before ICU admission available for Liabeuf et al. In Jung et al. mortality numbers were used as severe cases, as these were higher than those requiring mechanical ventilation. In Mehta et al, mortality data was only available for 211 ACEI/ARB, and 1494 in non-ACEI/ARB. In Cannata et al. 7 out of 39 who died continued ACEI/ARB during admission.

**Supp Table 4: Comparison between prescription of ACEI/ARB medication classes and associated severe COVID-19 and mortality**

|  | | | **Characteristics of people prescribed ARBs/ACEI (n=8389)** | | | | | | | **Characteristics of people not prescribed ARBs/ACEI (n=20989)** | | |
| --- | --- | --- | --- | --- | --- | --- | --- | --- | --- | --- | --- | --- |
| **Study** | **Country** | **Study design** | **ACEI/ ARB**  **(n=)** | **ACEI group** | **Severe or critical disease in ACEI** | **Mortality in ACEI** | **ARB group**  **(n=)** | **Severe or critical disease in ARB** | **Mortality in ARB** | **No ACEI/ARB**  **(n=)** | **Severe or critical disease No ACEI/ARB** | **Mortality**  **No ACEI/ARB** |
| Feng et al.  (2020) ^(1)^ | China | Retrospective cohort | 33 | 8 | 1 | NR | 27 | 4 | NR | 443 | 120 | NR |
| Gao et al.  (2020) ^(2)^ | China | Retrospective cohort | 183 | 52 | 24 | 1 | 131 | 52 | 3 | 2694 | 628 | 52 |
| Guo et al.  (2020) ^(3)^ | China | Retrospective cohort | 19 | NR | NR | NR | NR | NR | NR | 168 | 43 | 43 |
| Hu et al.  (2020) ^(4)^ | China | Retrospective cohort | 65 | NR | NR | NR | NR | NR | NR | 819 | 132 | 0 |
| Huang et al.  (2020) ^(5)^ | China | Retrospective cohort | 20 | NR | NR | NR | NR | NR | NR | 30 | 24 | 2 |
| Li & Wang et al.  (2020) ^(6)^ | China | Retrospective cohort | 115 | 35 | 16 | 7 | 83 | 43 | 15 | 247 | 116 | 56 |
| Li & Xu et al.  (2020) ^(7)^ | China | Ambispective  cohort | 42 | NR | NR | NR | NR | NR | NR | 506 | 250 | NR |
| Meng et al.  (2020) ^(8)^ | China | Retrospective cohort | 17 | 2 | 1 | 0 | 15 | 14 | 0 | 25 | 12 | 1 |
| Tan et al.  (2020) ^(9)^ | China | Retrospective cohort | 31 | 4 | NR | 0 | 27 | NR | 0 | 69 | 60 | 11 |
| Wang et al.  (2020) ^(10)^ | China | Retrospective cohort | 62 | 62 | 30 | 30 | NR | NR | NR | 282 | 103 | 103 |
| Yang et al.  (2020) ^(11)^ | China | Retrospective cohort | 43 | 43 | NR | NR | NR | NR | NR | 208 | 71 | 19 |
| Zhang et al.  (2020) ^(12)^ | China | Retrospective cohort | 188 | 31 | NR | NR | 157 | NR | NR | 940 | 92 | 92 |
| Zhou et al.  (2020) ^(13)^ | China | Retrospective cohort | 15 | NR | NR | NR | NR | NR | NR | 95 | 10 | 7 |
| Fosbol et al.  (2020) ^(14)^ | Denmark | Retrospective cohort | 895 | 377 | 90 | 76 | 530 | 110 | 84 | 3585 | 373 | 297 |
| Liabeuf et al.  (2020) ^(15)^ | France | Retrospective cohort | 96 | NR | NR | NR | NR | NR | NR | 172 | 64 | 30 |
| Cannata et al.  (2020) ^(16)^ | Italy | Prospective cohort | 173 | NR | NR | NR | NR | NR | NR | 224 | 39 | 39 |
| Conversano et al.  (2020) ^(17)^ | Italy | Retrospective cohort | 68 | 35 | 14 | 14 | 33 | 7 | 7 | 123 | 21 | 21 |
| Felice et al.  (2020) ^(18)^ | Italy | Retrospective cohort | 82 | 40 | 9 | 8 | 42 | 12 | 7 | 51 | 25 | 18 |
| Mancia et al.  (2020) ^(19)^ | Italy | Retrospective cohort | 2896 | 1502 | 197 | NR | 1394 | 167 | NR | 3376 | 253 | NR |
| Tedeschi et al.  (2020) ^(20)^ | Italy | Prospective cohort | 175 | 99 | 39 | 39 | 76 | 29 | 29 | 136 | 63 | 63 |
| Jung et al.  (2020) ^(21)^ | Korea | Retrospective cohort | 377 | 20 | NR | NR | 365 | NR | NR | 1577 | 51 | 51 |
| de Abajo et al.  (2020) ^(22)^ | Spain | Retrospective cohort | 477 | 240 | 101 | NR | 237 | 105 | NR | 662 | 187 | NR |
| Bean at al.  (2020) ^(23)^ | UK | Prospective cohort | 399 | 260 | NR | NR | 147 | NR | NR | 801 | 288 | 182 |
| Mehta et al.  (2020) ^(24)^ | USA | Retrospective cohort | 212 | 116 | 28 | NR | 98 | 20 | NR | 1523 | 228 | 34/1494 |
| Reynolds et al.  (2020) ^(25)^ | USA | Retrospective study | 1293 | 584 | 139 | NR | 739 | 187 | NR | 1280 | 315 | NR |
| Richardson et al.  (2020) ^(26)^ | USA | Prospective cohort | 413 | 168 | 55 | 55 | 245 | 75 | 75 | 953 | 254 | 254 |

**Legend:** Data are presented as number/total (percentage). NR= not reported. ACEI= angiotensin-converting enzyme inhibitor, ARB= angiotensin-2 receptor blocker. Reynolds et al. reported outcome data for 1293 people prescribed ACEI//ARB and 584 people prescribed ACEI and 739 people prescribed ARB separately. In Richardson et al. 140/168 people continued to take ACEI during hospitalisation and 194/245 people continued to take ARB. In Conversano, only data on the hypertensive group was available. For Mehta et al. out of 1705 patients with death status available, 42 deaths (2.5%) occurred. Eight of 211 patients (3.8%) were in the ACEI or ARB cohort and 34 of 1494 (2.1%) were in the no-ACEI or no-ARB cohort.

**Supp Table 5: Characteristics of people without a history of hypertension prescribed ACEI/ARB medication classes and associated severe COVID-19 and mortality based on available reported data**

| **Characteristics of people without hypertension using ARBs/ACEI (n=139)** | | | | | | | | | | | | | | | | | |
| --- | --- | --- | --- | --- | --- | --- | --- | --- | --- | --- | --- | --- | --- | --- | --- | --- | --- |
| **Study** | N | Age | Male Sex | DM | IHD | CHF | CKD | COPD | CCB | Diuretic | BB | AB | Systolic blood  pressure  (mm Hg) | Diastolic blood  pressure  (mm Hg) | C-reactive protein  (mg/L) | Severe or critical disease outcome | Mortality |
| Gao et al.  (2020) ^(2)^ | 17 | NR | NR | NR | NR | NR | NR | NR | NR | NR | NR | NR | NR | NR | NR | NR | NR |
| Jung et al.  (2020) ^(21)^ | 43 | NR | NR | NR | NR | NR | NR | NR | NR | NR | NR | NR | NR | NR | NR | NR | NR |
| Mehta et al.  (2020) ^(24)^ | 14 | NR | NR | NR | NR | NR | NR | NR | NR | NR | NR | NR | NR | NR | NR | NR | NR |
| Wang et al.  (2020) ^(10)^ | 79 | NR | NR | NR | NR | NR | NR | NR | NR | NR | NR | NR | NR | NR | NR | NR | NR |

**Legend:** Includes studies reporting outcomes in people with and without hypertension prescribed ACEI/ARB

**Supp Table 6: Characteristics of people with hypertension not prescribed ACEI/ARB medication classes and associated severe COVID-19 and mortality based on available reported or author provided data**

| **Characteristics of people with hypertension not using ARBs/ACEI (n=5496)** | | | | | | | | | | | | | | | | | |
| --- | --- | --- | --- | --- | --- | --- | --- | --- | --- | --- | --- | --- | --- | --- | --- | --- | --- |
| **Study** | N | Age | Male Sex | DM | IHD | CHF | CKD | COPD | CCB | Diuretic | BB | AB | Systolic blood  pressure  (mm Hg) | Diastolic blood  pressure  (mm Hg) | C-reactive protein  (mg/L) | Severe or critical disease outcome | Mortality |
| Feng et al.  (2020) ^(1)^ | 80 | NR | NR | NR | NR | NR | NR | NR | NR | NR | NR | NR | NR | NR | NR | 27 | NR |
| Gao et al.  (2020) ^(2)^ | 667 | NR | 339  (51%) | 177  (27%) | 110  (16%) | 9  (1%) | 16  (2%) | 10  (1%) | 492  (74%) | 23  (3%) | 100  (15%) | NR | NR | NR | NR | 30 | 30 |
| Hu et al.  (2020) ^(4)^ | 84 | 58  (52-67) | 48  (57%) | 14  (17%) | 5  (6%) | | 2  (5%) | 1  (1%) | 62  (74%) | NR | NR | NR | NR | NR | 16  (6-51) | 15 | 0 |
| Huang et al.  (2020) ^(5)^ | 30 | 67  (SD 13) | 17  (57%) | 49  (6%) | 13  (2%) | NR | NR | 0 | 29  (97%) | 1  (3%) | 7  (23%) | NR | 129  (SD 20) | 78  (SD 12) | 49  (7-125) | 24 | 2 |
| Li & Wang et al.  (2020) ^(6)^ | 247 | 67  (60-75) | 121  (49%) | 85  (34%) | 35  (14%) | 5  (2%) | 22  (9%) | 10  (4%) | NR | NR | NR | NR | NR | NR | 26  (4-60) | 116 | 56 |
| Meng et al.  (2020) ^(8)^ | 25 | 65  (55-68) | 15  (60%) | 4  (16%) | 6  (24%) | NR | NR | NR | 24  (96%) | 1  (4%) | 5  (20%) | 0 | NR | NR | NR | 12 | 1 |
| Tan et al.  (2020) [^(9)^] | 69 | 68  (57-71) | NR | 20  (29%) | 13  (19%) | NR | 5  (7%) | 7  (10%) | 24  (35%) | NR | 3  (4%) | 1  (1%) | NR | NR | 24  (7-63) | 60 | 11 |
| Yang et al.  (2020) [^(11)^] | 83 | 67  (62-75) | 41  (49%) | 25  (12%) | NR | 16  (8%) | 3  (4%) | 3  (4%) | NR | NR | NR | NR | 124  (120-143) | 75  (70-85) | 33.9  (5.1-119) | 35 | 11 |
| Zhang et al.^#^  (2020) [^(12)^] | 940 | 64  (57-69) | 503  (54%) | 118  (13%) | 56  (6%) | NR | 19  (2%) | 3  (0%) | 489  (52%) | 120  (13%) | 168  (18%) | 21  (2%) | 134  (121-145) | 80  (73-86) | >5  (69%) | 92 | 92 |
| Zhou et al.  (2020) [^(13)^] | 21 | 69  (SD 8) | 10  (48%) | NR | NR | NR | NR | NR | NR | NR | NR | NR | NR | NR | NR | 5 | 5 |
| Fosbol et al.  (2020) [^(14)^] | 196* / 209 | NR | NR | NR | NR | NR | NR | NR | NR | NR | NR | NR | NR | NR | NR | 37 | 36 |
| Liabeuf et al.  (2020) [^(15)^] | 56 | NR | NR | NR | NR | NR | NR | NR | NR | NR | NR | NR | NR | NR | NR | NR | NR |
| Felice et al.  (2020) [^(18)^] | 51 | 76  (SD 12) | 27  (53%) | 14  (28%) | 27  (53%) | | NR | 7  (14%) | NR | NR | NR | NR | 136  (SD 29) | 80  (SD 18) | NR | 25 | 18 |
| Tedeschi et al.  (2020) [^(20)^] | 136 | NR | 92  (68%) | 37  (27%) | 60  (44%) | NR | NR | 30  (22%) | 76  (56%) | | | | NR | NR | NR | 63 | 63 |
| Jung et al.  (2020) [^(21)^] | 438 | 64  (SD 12) | 215  (49%) | 187  (43%) | 19  (4%) | 81  (18%) | 88  (20%) | 203  (46%) | NR | NR | NR | NR | NR | NR | NR | 25 | 25 |
| de Abajo et al.  (2020) [^(22)^] | 140 | NR | NR | NR | NR | NR | NR | NR | NR | NR | NR | NR | NR | NR | NR | 64 | NR |
| Reynolds et al.  (2020) [^(25)^] | 1280 | NR | NR | NR | NR | NR | NR | NR | NR | NR | NR | NR | NR | NR | NR | 315 | NR |
| Richardson et al.  (2020) [^(26)^] | 953 | NR | NR | NR | NR | NR | NR | NR | NR | NR | NR | NR | NR | NR | NR | 254 | 254 |

**Legend:** Includes studies reporting outcomes in people with and without hypertension only.

* 196 CCB users in Fosbol et al. of 209 hypertensive non-ACEI/ARB.

**Supp Table 7:** **Assessment of study quality by independent assessors**

| **Questions** | | Feng et al.  (2020) ^(1)^ | | | Gao et al.  (2020) ^(2)^ | | | Guo et al.  (2020) ^(3)^ | | | Hu et al.  (2020) ^(4)^ | | | Huang et al.  (2020) ^(5)^ | | | Li & Wang et al. (2020) ^(6)^ | | Li & Xu et al.  (2020) ^(7)^ | | | Meng et al.  (2020) ^(8)^ | | | Tan et al.  (2020) ^(9)^ | | | Wang et al.  (2020) ^(10)^ | | | Yang et al.  (2020) ^(11)^ | | | | Zhang et al.  (2020) ^(12)^ | | | Zhou et al.  (2020) ^(13)^ | | | Fosbol et al.  (2020) ^(14)^ | | | Liabeuf et al.  (2020) ^(15)^ | | | Cannata et al.  (2020) ^(16)^ | | | Conversano et al. (2020) ^(17)^ | | | Felice et al.  (2020) ^(18)^ | | | Mancia et al.  (2020) ^(19)^ | | | Tedeschi et al.  (2020) ^(20)^ | | | Jung et al.  (2020) ^(21)^ | | | de Abajo et al.  (2020) ^(22)^ | | | Bean at al.  (2020) ^(23)^ | | | Mehta et al.  (2020) ^(24)^ | | | Reynolds et al.  (2020) ^(25)^ | | | Richardson et al. (2020) ^(26)^ | |
| --- | --- | --- | --- | --- | --- | --- | --- | --- | --- | --- | --- | --- | --- | --- | --- | --- | --- | --- | --- | --- | --- | --- | --- | --- | --- | --- | --- | --- | --- | --- | --- | --- | --- | --- | --- | --- | --- | --- | --- | --- | --- | --- | --- | --- | --- | --- | --- | --- | --- | --- | --- | --- | --- | --- | --- | --- | --- | --- | --- | --- | --- | --- | --- | --- | --- | --- | --- | --- | --- | --- | --- | --- | --- | --- | --- | --- | --- | --- |
| **Author Initial** | M  F | | A  D | M  F | | A  D | M  F | | A  D | M  F | | A  D | M  F | | A  D | M  F | | A  D | | M  F | A  D | | M  F | A  D | | M  F | A  D | | M  F | A  D | | M  F | A  D | M  F | | A  D | M  F | | A  D | M  F | | A  D | M  F | | A  D | M  F | | A  D | M  F | | A  D | M  F | | A  D | M  F | | A  D | M  F | | A  D | M  F | | A  D | M  F | | A  D | MF | | AD | M  F | | A  D | M  F | | A  D | M  F | | A  D |
| **Participant selection bias**  **Q1** | 0 | | 1 | 1 | | 1 | 0 | | 0 | 1 | | 1 | 0 | | 0 | 1 | | 1 | | 1 | 1 | | 1 | 1 | | 1 | 1 | | 0 | 0 | | 1 | 1 | 0 | | 0 | 0 | | 1 | 0 | | 1 | 1 | | 1 | 1 | | 1 | 0 | | 0 | 1 | | 1 | 1 | | 1 | 1 | | 1 | 0 | | 0 | 1 | | 1 | 1 | | 1 | 1 | | 1 | 1 | | 1 | 1 | | 1 |
| **Participant selection bias**  **Q2** | 1 | | 1 | 1 | | 1 | 1 | | 1 | 1 | | 1 | 1 | | 1 | 1 | | 1 | | 1 | 1 | | 1 | 1 | | 0 | 0 | | 1 | 1 | | 1 | 1 | 0 | | 0 | 0 | | 0 | 0 | | 1 | 1 | | 1 | 0 | | 0 | 0 | | 0 | 1 | | 1 | 1 | | 1 | 0 | | 0 | 1 | | 1 | 1 | | 1 | 1 | | 1 | 1 | | 1 | 1 | | 1 | 1 | | 1 |
| **Information bias**  **Q3** | 1 | | 1 | 1 | | 1 | 1 | | 1 | 1 | | 1 | 1 | | 1 | 0 | | 0 | | 1 | 1 | | 0 | 0 | | 1 | 1 | | 0 | 1 | | 1 | 0 | 1 | | 1 | 1 | | 1 | 1 | | 1 | 1 | | 0 | 0 | | 1 | 0 | | 1 | 0 | | 0 | 1 | | 1 | 1 | | 1 | 1 | | 1 | 0 | | 1 | 1 | | 1 | 0 | | 0 | 0 | | 0 | 1 | | 1 |
| **Information bias**  **Q4** | 1 | | 1 | 1 | | 1 | 0 | | 0 | 0 | | 0 | 1 | | 1 | 1 | | 1 | | 1 | 0 | | 0 | 0 | | 0 | 0 | | 0 | 1 | | 1 | 1 | 0 | | 0 | 0 | | 0 | 1 | | 1 | 0 | | 1 | 0 | | 0 | 0 | | 0 | 1 | | 0 | 0 | | 0 | 0 | | 0 | 0 | | 0 | 1 | | 1 | 1 | | 1 | 1 | | 1 | 1 | | 1 | 0 | | 0 |
| **Exposure definition**  **Q5** | 0 | | 0 | 1 | | 1 | 1 | | 1 | 1 | | 1 | 1 | | 1 | 1 | | 1 | | 1 | 1 | | 1 | 1 | | 0 | 0 | | 0 | 0 | | 1 | 1 | 1 | | 1 | 0 | | 0 | 0 | | 0 | 1 | | 1 | 1 | | 1 | 1 | | 1 | 1 | | 1 | 0 | | 0 | 0 | | 0 | 0 | | 0 | 0 | | 0 | 1 | | 1 | 0 | | 0 | 1 | | 1 | 0 | | 0 |
| **Confounding**  **Q6** | 0 | | 0 | 1 | | 1 | 0 | | 0 | 0 | | 0 | 0 | | 0 | 0 | | 0 | | 0 | 0 | | 0 | 0 | | 0 | 0 | | 0 | 0 | | 0 | 0 | 1 | | 1 | 0 | | 0 | 1 | | 1 | 1 | | 1 | 0 | | 0 | 0 | | 0 | 1 | | 1 | 1 | | 1 | 1 | | 1 | 1 | | 1 | 1 | | 0 | 1 | | 1 | 1 | | 1 | 1 | | 1 | 0 | | 0 |
| **Confounding**  **Q7** | 0 | | 0 | 1 | | 1 | 0 | | 0 | 0 | | 0 | 0 | | 0 | 0 | | 0 | | 0 | 0 | | 0 | 0 | | 0 | 0 | | 0 | 0 | | 0 | 0 | 1 | | 1 | 0 | | 0 | 1 | | 1 | 0 | | 1 | 1 | | 1 | 0 | | 0 | 0 | | 1 | 1 | | 1 | 1 | | 1 | 0 | | 0 | 0 | | 0 | 1 | | 1 | 0 | | 0 | 1 | | 1 | 0 | | 1 |
| **Information bias**  **Q8** | 0 | | 1 | 0 | | 1 | 0 | | 0 | 0 | | 0 | 1 | | 1 | 0 | | 0 | | 0 | 1 | | 1 | 0 | | 0 | 0 | | 0 | 0 | | 0 | 0 | 1 | | 1 | 0 | | 0 | 0 | | 0 | 0 | | 0 | 0 | | 0 | 0 | | 1 | 0 | | 0 | 0 | | 0 | 0 | | 0 | 0 | | 0 | 0 | | 1 | 1 | | 1 | 0 | | 0 | 1 | | 1 | 0 | | 0 |
| **Information bias**  **Q9** | 1 | | 1 | 1 | | 0 | 0 | | 0 | 1 | | 1 | 1 | | 1 | 0 | | 0 | | 0 | 0 | | 0 | 0 | | 1 | 1 | | 1 | 1 | | 0 | 0 | 0 | | 0 | 1 | | 1 | 0 | | 0 | 1 | | 1 | 1 | | 1 | 0 | | 0 | 0 | | 1 | 1 | | 1 | 1 | | 1 | 0 | | 0 | 1 | | 0 | 0 | | 0 | 1 | | 1 | 1 | | 1 | 0 | | 0 |
| **Precision**  **Q10** | 0 | | 0 | 1 | | 1 | 0 | | 0 | 0 | | 0 | 0 | | 0 | 0 | | 0 | | 0 | 0 | | 0 | 0 | | 0 | 0 | | 0 | 0 | | 0 | 0 | 1 | | 1 | 0 | | 0 | 1 | | 1 | 1 | | 1 | 1 | | 1 | 0 | | 0 | 1 | | 1 | 1 | | 1 | 1 | | 1 | 1 | | 1 | 0 | | 0 | 1 | | 1 | 1 | | 1 | 1 | | 1 | 0 | | 0 |
| **Totals** | 4 | | 6 | 9 | | 9 | 3 | | 3 | 5 | | 5 | 6 | | 6 | 4 | | 4 | | 5 | 5 | | 4 | 3 | | 3 | 3 | | 2 | 4 | | 5 | 4 | 6 | | 6 | 2 | | 3 | 5 | | 7 | 7 | | 8 | 5 | | 6 | 1 | | 3 | 6 | | 7 | 7 | | 7 | 6 | | 6 | 4 | | 4 | 5 | | 5 | 9 | | 9 | 6 | | 6 | 9 | | 9 | 3 | | 4 |
| **Consensus percentage** | **60%** | | | **90%** | | | **30%** | | | **50%** | | | **60%** | | | **40%** | | | | **50%** | | | **40%** | | | **30%** | | | **40%** | | | **40%** | | **60%** | | | **20%** | | | **50%** | | | **70%** | | | **60%** | | | **30%** | | | **60%** | | | **70%** | | | **60%** | | | **40%** | | | **50%** | | | **90%** | | | **60%** | | | **90%** | | | **30%** | | |

**Legend:** Q1) Did the number of people in the cohort represent the number of eligible people? Q2) Did Method of COVID-19 diagnosis meet guidelines? Q3) Was a standardized definition of death (outcome) given? Q4) Was a standardized definition of disease severity given? Q5) Was ACE/ARB use recorded on hospital admission? Q6) Did the study adjust for age and sex in antihypertensive specific severity AND/OR mortality outcomes? Q7) Did the study adjust for diabetes, CKD, IHD in antihypertensive specific severity AND/OR mortality outcomes? Q8) Did the study follow-up patients until hospital discharge/death? Q9) Was there less than 10% missing data in relation to antihypertensive specific severity AND/OR mortality outcomes? Q10) Did the study report confidence intervals or a precision estimate of effect of ACE/ARB with severity AND/OR mortality outcomes? MF and AD were the two independent raters. The total scores from independent review and the final agreed percentage out of 100 is reported.

**Supp Table 8:** **Sensitivity analysis from Meta-analysis on severe or critical disease outcome of COVID-19**

| **Comparison** | **Pooled effect size** | **Heterogeneity** | **Statistical significance** |
| --- | --- | --- | --- |
| **Overall effect** | 1.23 [1.06, 1.42] | Heterogeneity: Tau² = 0.11; Chi² = 200.93, df = 25 (P < 0.00001); I² = 88% | Test for overall effect: Z = 2.73 (P = 0.006) |
| **Studies from Asia** | 1.19 [0.94, 1.51] | Heterogeneity: Tau² = 0.14; Chi² = 79.17, df = 13 (P < 0.00001); I² = 84% | Test for overall effect: Z = 1.48 (P = 0.14) |
| **Studies from the USA** | 1.17 [0.96, 1.43] | Heterogeneity: Tau² = 0.02; Chi² = 6.76, df = 2 (P = 0.03); I² = 70% | Test for overall effect: Z = 1.54 (P = 0.12) |
| **Studies from Europe** | 1.27 [0.97, 1.65] | Heterogeneity: Tau² = 0.14; Chi² = 99.41, df = 8 (P < 0.00001); I² = 92% | Test for overall effect: Z = 1.75 (P = 0.08) |
| **Removing possible dual reporting in multiple studies** | | | |
| **Excluding:**  Guo et al.  (2020) ^(3)^  Zhang et al.  (2020) ^(12)^ | 1.25 [1.08, 1.46] | Heterogeneity: Tau² = 0.11; Chi² = 192.89, df = 23 (P < 0.00001); I² = 88% | Test for overall effect: Z = 2.95 (P = 0.003) |
| **Excluding:**  Huang et al.  (2020) ^(5)^  Zhang et al.  (2020) ^(12)^ | 1.28 [1.10, 1.49] | Heterogeneity: Tau² = 0.11; Chi² = 186.27, df = 23 (P < 0.00001); I² = 88% | Test for overall effect: Z = 3.26 (P = 0.001) |
| **Excluding:**  Li & Xu et al.  (2020) ^(7)^  Wang et al.  (2020) ^(10)^ | 1.24 [1.06, 1.45] | Heterogeneity: Tau² = 0.11; Chi² = 196.89, df = 23 (P < 0.00001); I² = 88% | Test for overall effect: Z = 2.68 (P = 0.007) |
| **Excluding:**  Guo et al.  (2020) ^(3)^  Zhang et al.  (2020) ^(12)^  Huang et al.  (2020) ^(5)^  Wang et al.  (2020) ^(10)^  Li & Xu et al.  (2020) ^(7)^ | 1.30 [1.10, 1.52] | Heterogeneity: Tau² = 0.11; Chi² = 181.87, df = 20 (P < 0.00001); I² = 89% | Test for overall effect: Z = 3.12 (P = 0.002) |
| **High quality studies**  Gao et al.  (2020) ^(2)^  Bean at al.  (2020) ^(23)^  Reynolds et al.  (2020) ^(25)^ | 1.15 [0.79, 1.67] | Heterogeneity: Tau² = 0.10; Chi² = 30.98, df = 2 (P < 0.00001); I² = 94% | Test for overall effect: Z = 0.74 (P = 0.46) |
| **Leave one out analysis**  **Excluding:** | | | |
| Feng et al.  (2020) ^(1)^ | 1.25 [1.08, 1.45] | Heterogeneity: Tau² = 0.11; Chi² = 196.07, df = 24 (P < 0.00001); I² = 88% | Test for overall effect: Z = 2.96 (P = 0.003) |
| Gao et al.  (2020) ^(2)^ | 1.21 [1.04, 1.41] | Heterogeneity: Tau² = 0.11; Chi² = 191.48, df = 24 (P < 0.00001); I² = 87% | Test for overall effect: Z = 2.41 (P = 0.02) |
| Guo et al.  (2020) ^(3)^ | 1.22 [1.05, 1.42] | Heterogeneity: Tau² = 0.11; Chi² = 200.83, df = 24 (P < 0.00001); I² = 88% | Test for overall effect: Z = 2.62 (P = 0.009) |
| Hu et al.  (2020) ^(4)^ | 1.19 [1.03, 1.38] | Heterogeneity: Tau² = 0.10; Chi² = 181.21, df = 24 (P < 0.00001); I² = 87% | Test for overall effect: Z = 2.35 (P = 0.02) |
| Huang et al.  (2020) ^(5)^ | 1.25 [1.08, 1.45] | Heterogeneity: Tau² = 0.11; Chi² = 194.44, df = 24 (P < 0.00001); I² = 88% | Test for overall effect: Z = 2.91 (P = 0.004) |
| Li & Wang et al.  (2020) ^(6)^ | 1.23 [1.05, 1.43] | Heterogeneity: Tau² = 0.12; Chi² = 200.70, df = 24 (P < 0.00001); I² = 88% | Test for overall effect: Z = 2.57 (P = 0.01) |
| Li & Xu et al.  (2020) ^(7)^ | 1.24 [1.07, 1.45] | Heterogeneity: Tau² = 0.11; Chi² = 196.89, df = 24 (P < 0.00001); I² = 88% | Test for overall effect: Z = 2.83 (P = 0.005) |
| Meng et al.  (2020) ^(8)^ | 1.25 [1.08, 1.45] | Heterogeneity: Tau² = 0.11; Chi² = 196.92, df = 24 (P < 0.00001); I² = 88% | Test for overall effect: Z = 2.93 (P = 0.003) |
| Tan et al.  (2020) ^(9)^ | 1.24 [1.06, 1.45] | Heterogeneity: Tau² = 0.11; Chi² = 190.08, df = 24 (P < 0.00001); I² = 87% | Test for overall effect: Z = 2.75 (P = 0.006) |
| Wang et al.  (2020) ^(10)^ | 1.22 [1.05, 1.43] | Heterogeneity: Tau² = 0.11; Chi² = 200.92, df = 24 (P < 0.00001); I² = 88% | Test for overall effect: Z = 2.58 (P = 0.010) |
| Yang et al.  (2020) ^(11)^ | 1.24 [1.06, 1.44] | Heterogeneity: Tau² = 0.11; Chi² = 199.84, df = 24 (P < 0.00001); I² = 88% | Test for overall effect: Z = 2.75 (P = 0.006) |
| Zhang et al.  (2020) ^(12)^ | 1.26 [1.09, 1.46] | Heterogeneity: Tau² = 0.11; Chi² = 192.97, df = 24 (P < 0.00001); I² = 88% | Test for overall effect: Z = 3.06 (P = 0.002) |
| Zhou et al.  (2020) ^(13)^ | 1.22 [1.05, 1.42] | Heterogeneity: Tau² = 0.11; Chi² = 200.52, df = 24 (P < 0.00001); I² = 88% | Test for overall effect: Z = 2.64 (P = 0.008) |
| Fosbol et al.  (2020) ^(14)^ | 1.20 [1.04, 1.38] | Heterogeneity: Tau² = 0.09; Chi² = 152.87, df = 24 (P < 0.00001); I² = 84% | Test for overall effect: Z = 2.54 (P = 0.01) |
| Liabeuf et al.  (2020) ^(15)^ | 1.22 [1.04, 1.42] | Heterogeneity: Tau² = 0.11; Chi² = 200.23, df = 24 (P < 0.00001); I² = 88% | Test for overall effect: Z = 2.52 (P = 0.01) |
| Cannata et al.  (2020) ^(16)^ | 1.23 [1.05, 1.43] | Heterogeneity: Tau² = 0.11; Chi² = 200.93, df = 24 (P < 0.00001); I² = 88% | Test for overall effect: Z = 2.62 (P = 0.009) |
| Conversano et al.  (2020) ^(17)^ | 1.21 [1.04, 1.41] | Heterogeneity: Tau² = 0.11; Chi² = 199.37, df = 24 (P < 0.00001); I² = 88% | Test for overall effect: Z = 2.52 (P = 0.01) |
| Felice et al.  (2020) ^(18)^ | 1.27 [1.10, 1.47] | Heterogeneity: Tau² = 0.10; Chi² = 185.96, df = 24 (P < 0.00001); I² = 87% | Test for overall effect: Z = 3.19 (P = 0.001) |
| Mancia et al.  (2020) ^(19)^ | 1.21 [1.04, 1.41] | Heterogeneity: Tau² = 0.11; Chi² = 188.54, df = 24 (P < 0.00001); I² = 87% | Test for overall effect: Z = 2.41 (P = 0.02) |
| Tedeschi et al.  (2020) ^(20)^ | 1.25 [1.08, 1.45] | Heterogeneity: Tau² = 0.11; Chi² = 189.64, df = 24 (P < 0.00001); I² = 87% | Test for overall effect: Z = 2.93 (P = 0.003) |
| Jung et al.  (2020) ^(21)^ | 1.19 [1.03, 1.38] | Heterogeneity: Tau² = 0.11; Chi² = 189.09, df = 24 (P < 0.00001); I² = 87% | Test for overall effect: Z = 2.36 (P = 0.02) |
| de Abajo et al.  (2020) ^(22)^ | 1.21 [1.04, 1.42] | Heterogeneity: Tau² = 0.12; Chi² = 196.47, df = 24 (P < 0.00001); I² = 88% | Test for overall effect: Z = 2.42 (P = 0.02) |
| Bean at al.  (2020) ^(23)^ | 1.25 [1.08, 1.45] | Heterogeneity: Tau² = 0.11; Chi² = 180.30, df = 24 (P < 0.00001); I² = 87% | Test for overall effect: Z = 2.93 (P = 0.003) |
| Mehta et al.  (2020) ^(24)^ | 1.22 [1.04, 1.42] | Heterogeneity: Tau² = 0.11; Chi² = 200.03, df = 24 (P < 0.00001); I² = 88% | Test for overall effect: Z = 2.52 (P = 0.01) |
| Reynolds et al.  (2020) ^(25)^ | 1.24 [1.06, 1.45] | Heterogeneity: Tau² = 0.12; Chi² = 185.62, df = 24 (P < 0.00001); I² = 87% | Test for overall effect: Z = 2.73 (P = 0.006) |
| Richardson et al.  (2020) ^(26)^ | 1.23 [1.05, 1.44] | Heterogeneity: Tau² = 0.12; Chi² = 199.83, df = 24 (P < 0.00001); I² = 88% | Test for overall effect: Z = 2.58 (P = 0.010) |

**Supp Table 9:** **Sensitivity analysis from Meta-analysis on severe or critical disease outcome of COVID-19 in people with a history of hypertension**

| **Comparison** | **Pooled effect size** | **Heterogeneity** | **Statistical significance** |
| --- | --- | --- | --- |
| **Overall effect** | 0.91 [0.74, 1.11] | Heterogeneity: Tau² = 0.07; Chi² = 40.16, df = 11 (P < 0.0001); I² = 73% | Test for overall effect: Z = 0.97 (P = 0.33) |
| **Studies from Asia** | 0.92 [0.70, 1.20] | Heterogeneity: Tau² = 0.09; Chi² = 27.69, df = 8 (P = 0.0005); I² = 71% | Test for overall effect: Z = 0.62 (P = 0.54) |
| **Studies from the USA** | 1.18 [0.99, 1.41] | Heterogeneity: Not applicable | Test for overall effect: Z = 1.84 (P = 0.07) |
| **Studies from Europe** | 0.69 [0.44, 1.09] | Heterogeneity: Tau² = 0.08; Chi² = 3.06, df = 1 (P = 0.08); I² = 67% | Test for overall effect: Z = 1.59 (P = 0.11) |
| **Removing possible dual reporting in multiple studies** | | | |
| **Excluding:**  Huang et al.  (2020) ^(5)^  Zhang et al.  (2020) ^(12)^ | 0.96 [0.77, 1.19] | Heterogeneity: Tau² = 0.07; Chi² = 33.64, df = 9 (P = 0.0001); I² = 73% | Test for overall effect: Z = 0.40 (P = 0.69) |
| **Leave one out analysis**  **Excluding:** | | | |
| Feng et al.  (2020) ^(1)^ | 0.94 [0.77, 1.14] | Heterogeneity: Tau² = 0.06; Chi² = 35.55, df = 10 (P = 0.0001); I² = 72% | Test for overall effect: Z = 0.64 (P = 0.52) |
| Hu et al.  (2020) ^(4)^ | 0.86 [0.71, 1.03] | Heterogeneity: Tau² = 0.05; Chi² = 30.36, df = 10 (P = 0.0007); I² = 67% | Test for overall effect: Z = 1.63 (P = 0.10) |
| Huang et al.  (2020) ^(5)^ | 0.91 [0.74, 1.13] | Heterogeneity: Tau² = 0.08; Chi² = 38.77, df = 10 (P < 0.0001); I² = 74% | Test for overall effect: Z = 0.83 (P = 0.41) |
| Li & Wang et al.  (2020) ^(6)^ | 0.86 [0.69, 1.07] | Heterogeneity: Tau² = 0.08; Chi² = 35.60, df = 10 (P < 0.0001); I² = 72% | Test for overall effect: Z = 1.33 (P = 0.18) |
| Meng et al.  (2020) ^(8)^ | 0.93 [0.76, 1.13] | Heterogeneity: Tau² = 0.07; Chi² = 37.81, df = 10 (P < 0.0001); I² = 74% | Test for overall effect: Z = 0.74 (P = 0.46) |
| Tan et al.  (2020) ^(9)^ | 0.87 [0.68, 1.12] | Heterogeneity: Tau² = 0.10; Chi² = 40.16, df = 10 (P < 0.0001); I² = 75% | Test for overall effect: Z = 1.07 (P = 0.28) |
| Yang et al.  (2020) ^(11)^ | 0.91 [0.73, 1.13] | Heterogeneity: Tau² = 0.07; Chi² = 39.40, df = 10 (P < 0.0001); I² = 75% | Test for overall effect: Z = 0.87 (P = 0.38) |
| Zhang et al.  (2020) ^(12)^ | 0.94 [0.77, 1.15] | Heterogeneity: Tau² = 0.06; Chi² = 35.31, df = 10 (P = 0.0001); I² = 72% | Test for overall effect: Z = 0.58 (P = 0.56) |
| Zhou et al.  (2020) ^(13)^ | 0.90 [0.74, 1.11] | Heterogeneity: Tau² = 0.07; Chi² = 40.06, df = 10 (P < 0.0001); I² = 75% | Test for overall effect: Z = 0.95 (P = 0.34) |
| Felice et al.  (2020) ^(18)^ | 0.96 [0.79, 1.17] | Heterogeneity: Tau² = 0.06; Chi² = 31.85, df = 10 (P = 0.0004); I² = 69% | Test for overall effect: Z = 0.41 (P = 0.68) |
| Tedeschi et al.  (2020) ^(20)^ | 0.91 [0.73, 1.14] | Heterogeneity: Tau² = 0.08; Chi² = 37.75, df = 10 (P < 0.0001); I² = 74% | Test for overall effect: Z = 0.84 (P = 0.40) |
| Richardson et al.  (2020) ^(26)^ | 0.86 [0.68, 1.09] | Heterogeneity: Tau² = 0.09; Chi² = 37.70, df = 10 (P < 0.0001); I² = 73% | Test for overall effect: Z = 1.27 (P = 0.21) |

**Supp Table 10:** **Sensitivity analysis from Meta-analysis on severe or critical disease outcome of COVID-19 comparison between ACEI vs. No ACEI or ARB and ARB vs. No ARB or ACEI**

|  | **ACEI vs. No ACEI or ARB** | | | **ARB vs. no ARB or ACEI** | | |
| --- | --- | --- | --- | --- | --- | --- |
| **Comparison/ Studies excluded** | **Pooled effect size** | **Heterogeneity** | **Statistical significance** | **Pooled effect size** | **Heterogeneity** | **Statistical significance** |
| **Overall effect** | 1.33 [1.08, 1.63] | Heterogeneity: Tau² = 0.11; Chi² = 83.80, df = 13 (P < 0.00001); I² = 84% | Test for overall effect: Z = 2.68 (P = 0.007) | 1.28 [1.07, 1.52] | Heterogeneity: Tau² = 0.08; Chi² = 65.80, df = 12 (P < 0.00001); I² = 82% | Test for overall effect: Z = 2.68 (P = 0.007) |
| **Studies from Asia** | 1.31 [0.91, 1.89] | Heterogeneity: Tau² = 0.09; Chi² = 10.52, df = 4 (P = 0.03); I² = 62% | Test for overall effect: Z = 1.46 (P = 0.14) | 1.35 [0.93, 1.96] | Heterogeneity: Tau² = 0.10; Chi² = 13.25, df = 3 (P = 0.004); I² = 77% | Test for overall effect: Z = 1.57 (P = 0.12) |
| **Studies from the USA** | 1.20 [0.91, 1.59] | Heterogeneity: Tau² = 0.04; Chi² = 7.65, df = 2 (P = 0.02); I² = 74% | Test for overall effect: Z = 1.31 (P = 0.19) | 1.09 [0.97, 1.23] | Heterogeneity: Tau² = 0.00; Chi² = 1.91, df = 2 (P = 0.38); I² = 0% | Test for overall effect: Z = 1.41 (P = 0.16) |
| **Studies from Europe** | 1.40 [1.00, 1.96] | Heterogeneity: Tau² = 0.14; Chi² = 46.48, df = 5 (P < 0.00001); I² = 89% | Test for overall effect: Z = 1.94 (P = 0.05) | 1.28 [0.96, 1.72] | Heterogeneity: Tau² = 0.10; Chi² = 32.39, df = 5 (P < 0.00001); I² = 85% | Test for overall effect: Z = 1.69 (P = 0.09) |
| **High quality studies**  Gao et al.  (2020) ^(2)^  Reynolds et al.  (2020) ^(25)^ | 1.37 [0.67, 2.82] | Heterogeneity: Tau² = 0.25; Chi² = 17.16, df = 1 (P < 0.0001); I² = 94% | Test for overall effect: Z = 0.86 (P = 0.39) | 1.32 [0.80, 2.17] | Heterogeneity: Tau² = 0.12; Chi² = 13.69, df = 1 (P = 0.0002); I² = 93% | Test for overall effect: Z = 1.07 (P = 0.29) |
| **Leave one out analysis**  **Excluding:** | | | | | | |
| Feng et al.  (2020) ^(1)^ | 1.34 [1.09, 1.66] | Heterogeneity: Tau² = 0.11; Chi² = 82.34, df = 12 (P < 0.00001); I² = 85% | Test for overall effect: Z = 2.79 (P = 0.005) | 1.31 [1.10, 1.56] | Heterogeneity: Tau² = 0.07; Chi² = 61.95, df = 11 (P < 0.00001); I² = 82% | Test for overall effect: Z = 2.97 (P = 0.003) |
| Gao et al.  (2020) ^(2)^ | 1.28 [1.03, 1.59] | Heterogeneity: Tau² = 0.11; Chi² = 78.46, df = 12 (P < 0.00001); I² = 85% | Test for overall effect: Z = 2.23 (P = 0.03) | 1.24 [1.02, 1.50] | Heterogeneity: Tau² = 0.08; Chi² = 61.10, df = 11 (P < 0.00001); I² = 82% | Test for overall effect: Z = 2.18 (P = 0.03) |
| Li & Wang et al.  (2020) ^(6)^ | 1.36 [1.10, 1.69] | Heterogeneity: Tau² = 0.11; Chi² = 80.18, df = 12 (P < 0.00001); I² = 85% | Test for overall effect: Z = 2.80 (P = 0.005) | 1.29 [1.07, 1.56] | Heterogeneity: Tau² = 0.08; Chi² = 63.08, df = 11 (P < 0.00001); I² = 83% | Test for overall effect: Z = 2.61 (P = 0.009) |
| Meng et al.  (2020) ^(8)^ | 1.33 [1.08, 1.65] | Heterogeneity: Tau² = 0.11; Chi² = 83.64, df = 12 (P < 0.00001); I² = 86% | Test for overall effect: Z = 2.68 (P = 0.007) | 1.24 [1.03, 1.49] | Heterogeneity: Tau² = 0.08; Chi² = 62.88, df = 11 (P < 0.00001); I² = 83% | Test for overall effect: Z = 2.27 (P = 0.02) |
| Wang et al.  (2020) ^(10)^ | 1.33 [1.06, 1.66] | Heterogeneity: Tau² = 0.12; Chi² = 83.66, df = 12 (P < 0.00001); I² = 86% | Test for overall effect: Z = 2.46 (P = 0.01) | NR | NR | NR |
| Fosbol et al.  (2020) ^(14)^ | 1.26 [1.04, 1.54] | Heterogeneity: Tau² = 0.09; Chi² = 58.59, df = 12 (P < 0.00001); I² = 80% | Test for overall effect: Z = 2.34 (P = 0.02) | 1.22 [1.03, 1.45] | Heterogeneity: Tau² = 0.06; Chi² = 47.77, df = 11 (P < 0.00001); I² = 77% | Test for overall effect: Z = 2.29 (P = 0.02) |
| Conversano et al.  (2020) ^(17)^ | 1.28 [1.04, 1.59] | Heterogeneity: Tau² = 0.11; Chi² = 80.59, df = 12 (P < 0.00001); I² = 85% | Test for overall effect: Z = 2.30 (P = 0.02) | 1.28 [1.06, 1.53] | Heterogeneity: Tau² = 0.08; Chi² = 65.75, df = 11 (P < 0.00001); I² = 83% | Test for overall effect: Z = 2.60 (P = 0.009) |
| Felice et al.  (2020) ^(18)^ | 1.41 [1.15, 1.72] | Heterogeneity: Tau² = 0.10; Chi² = 71.75, df = 12 (P < 0.00001); I² = 83% | Test for overall effect: Z = 3.36 (P = 0.0008) | 1.34 [1.12, 1.59] | Heterogeneity: Tau² = 0.07; Chi² = 56.77, df = 11 (P < 0.00001); I² = 81% | Test for overall effect: Z = 3.28 (P = 0.001) |
| Mancia et al.  (2020) ^(19)^ | 1.29 [1.02, 1.62] | Heterogeneity: Tau² = 0.13; Chi² = 77.37, df = 12 (P < 0.00001); I² = 84% | Test for overall effect: Z = 2.16 (P = 0.03) | 1.24 [1.02, 1.51] | Heterogeneity: Tau² = 0.09; Chi² = 62.48, df = 11 (P < 0.00001); I² = 82% | Test for overall effect: Z = 2.15 (P = 0.03) |
| Tedeschi et al.  (2020) ^(20)^ | 1.39 [1.12, 1.71] | Heterogeneity: Tau² = 0.10; Chi² = 72.65, df = 12 (P < 0.00001); I² = 83% | Test for overall effect: Z = 3.06 (P = 0.002) | 1.33 [1.11, 1.59] | Heterogeneity: Tau² = 0.07; Chi² = 57.24, df = 11 (P < 0.00001); I² = 81% | Test for overall effect: Z = 3.11 (P = 0.002) |
| de Abajo et al.  (2020) ^(22)^ | 1.31 [1.03, 1.65] | Heterogeneity: Tau² = 0.13; Chi² = 83.42, df = 12 (P < 0.00001); I² = 86% | Test for overall effect: Z = 2.24 (P = 0.03) | 1.24 [1.02, 1.51] | Heterogeneity: Tau² = 0.09; Chi² = 62.96, df = 11 (P < 0.00001); I² = 83% | Test for overall effect: Z = 2.16 (P = 0.03) |
| Mehta et al.  (2020) ^(24)^ | 1.30 [1.05, 1.63] | Heterogeneity: Tau² = 0.12; Chi² = 83.19, df = 12 (P < 0.00001); I² = 86% | Test for overall effect: Z = 2.35 (P = 0.02) | 1.27 [1.05, 1.53] | Heterogeneity: Tau² = 0.08; Chi² = 65.80, df = 11 (P < 0.00001); I² = 83% | Test for overall effect: Z = 2.46 (P = 0.01) |
| Reynolds et al.  (2020) ^(25)^ | 1.38 [1.12, 1.69] | Heterogeneity: Tau² = 0.10; Chi² = 61.79, df = 12 (P < 0.00001); I² = 81% | Test for overall effect: Z = 3.07 (P = 0.002) | 1.31 [1.09, 1.57] | Heterogeneity: Tau² = 0.07; Chi² = 51.07, df = 11 (P < 0.00001); I² = 78% | Test for overall effect: Z = 2.89 (P = 0.004) |
| Richardson et al.  (2020) ^(26)^ | 1.33 [1.06, 1.67] | Heterogeneity: Tau² = 0.12; Chi² = 82.46, df = 12 (P < 0.00001); I² = 85% | Test for overall effect: Z = 2.49 (P = 0.01) | 1.29 [1.06, 1.56] | Heterogeneity: Tau² = 0.08; Chi² = 63.36, df = 11 (P < 0.00001); I² = 83% | Test for overall effect: Z = 2.53 (P = 0.01) |

**Supp Table 11:** **Sensitivity analysis from Meta-analysis on mortality from COVID-19**

| **Comparison** | **Pooled effect size** | **Heterogeneity** | **Statistical significance** |
| --- | --- | --- | --- |
| **Overall effect** | 1.18 [0.92, 1.50] | Heterogeneity: Tau² = 0.19; Chi² = 114.17, df = 20 (P < 0.00001); I² = 82% | Test for overall effect: Z = 1.31 (P = 0.19) |
| **Studies from Asia** | 1.07 [0.67, 1.71] | Heterogeneity: Tau² = 0.34; Chi² = 39.03, df = 11 (P < 0.0001); I² = 72% | Test for overall effect: Z = 0.29 (P = 0.77) |
| **Studies from the USA** | 1.18 [0.64, 2.17] | Heterogeneity: Tau² = 0.14; Chi² = 3.97, df = 2 (P = 0.14); I² = 50% | Test for overall effect: Z = 0.54 (P = 0.59) |
| **Studies from Europe** | 1.14 [0.76, 1.71] | Heterogeneity: Tau² = 0.27; Chi² = 75.45, df = 7 (P < 0.00001); I² = 91% | Test for overall effect: Z = 0.61 (P = 0.54) |
| **Removing possible dual reporting in multiple studies** | | | |
| **Excluding:**  Guo et al.  (2020) ^(3)^  Zhang et al.  (2020) ^(12)^ | 1.23 [0.96, 1.59] | Heterogeneity: Tau² = 0.18; Chi² = 102.72, df = 18 (P < 0.00001); I² = 82% | Test for overall effect: Z = 1.65 (P = 0.10) |
| **Excluding:**  Huang et al.  (2020) ^(5)^  Zhang et al.  (2020) ^(12)^ | 1.26 [0.99, 1.60] | Heterogeneity: Tau² = 0.17; Chi² = 101.70, df = 18 (P < 0.00001); I² = 82% | Test for overall effect: Z = 1.86 (P = 0.06) |
| **Excluding:**  Guo et al.  (2020) ^(3)^  Zhang et al.  (2020) ^(12)^  Huang et al.  (2020) ^(5)^  Wang et al.  (2020) ^(10)^ | 1.24 [0.94, 1.63] | Heterogeneity: Tau² = 0.20; Chi² = 101.60, df = 16 (P < 0.00001); I² = 84% | Test for overall effect: Z = 1.52 (P = 0.13) |
| **High quality studies**  Gao et al.  (2020) ^(2)^  Bean at al.  (2020) ^(23)^ | 1.17 [0.95, 1.43] | Heterogeneity: Tau² = 0.00; Chi² = 0.00, df = 1 (P = 0.95); I² = 0% | Test for overall effect: Z = 1.50 (P = 0.13) |
| **Leave one out analysis**  **Excluding:** | | | |
| Gao et al.  (2020) ^(2)^ | 1.18 [0.92, 1.51] | Heterogeneity: Tau² = 0.19; Chi² = 114.03, df = 19 (P < 0.00001); I² = 83% | Test for overall effect: Z = 1.28 (P = 0.20) |
| Guo et al.  (2020) ^(3)^ | 1.16 [0.90, 1.50] | Heterogeneity: Tau² = 0.20; Chi² = 114.14, df = 19 (P < 0.00001); I² = 83% | Test for overall effect: Z = 1.16 (P = 0.25) |
| Hu et al.  (2020) ^(4)^ | 1.16 [0.91, 1.47] | Heterogeneity: Tau² = 0.18; Chi² = 110.05, df = 19 (P < 0.00001); I² = 83% | Test for overall effect: Z = 1.18 (P = 0.24) |
| Huang et al.  (2020) ^(5)^ | 1.19 [0.93, 1.52] | Heterogeneity: Tau² = 0.19; Chi² = 113.14, df = 19 (P < 0.00001); I² = 83% | Test for overall effect: Z = 1.37 (P = 0.17) |
| Li & Wang et al.  (2020) ^(6)^ | 1.21 [0.94, 1.55] | Heterogeneity: Tau² = 0.19; Chi² = 108.67, df = 19 (P < 0.00001); I² = 83% | Test for overall effect: Z = 1.47 (P = 0.14) |
| Meng et al.  (2020) ^(8)^ | 1.18 [0.93, 1.51] | Heterogeneity: Tau² = 0.19; Chi² = 113.74, df = 19 (P < 0.00001); I² = 83% | Test for overall effect: Z = 1.34 (P = 0.18) |
| Tan et al.  (2020) ^(9)^ | 1.20 [0.94, 1.53] | Heterogeneity: Tau² = 0.18; Chi² = 110.55, df = 19 (P < 0.00001); I² = 83% | Test for overall effect: Z = 1.46 (P = 0.14) |
| Wang et al.  (2020) ^(10)^ | 1.16 [0.89, 1.51] | Heterogeneity: Tau² = 0.21; Chi² = 114.16, df = 19 (P < 0.00001); I² = 83% | Test for overall effect: Z = 1.11 (P = 0.27) |
| Yang et al.  (2020) ^(11)^ | 1.20 [0.94, 1.53] | Heterogeneity: Tau² = 0.19; Chi² = 112.26, df = 19 (P < 0.00001); I² = 83% | Test for overall effect: Z = 1.44 (P = 0.15) |
| Zhang et al.  (2020) ^(12)^ | 1.25 [0.98, 1.59] | Heterogeneity: Tau² = 0.17; Chi² = 102.73, df = 19 (P < 0.00001); I² = 82% | Test for overall effect: Z = 1.79 (P = 0.07) |
| Zhou et al.  (2020) ^(13)^ | 1.17 [0.91, 1.49] | Heterogeneity: Tau² = 0.19; Chi² = 114.03, df = 19 (P < 0.00001); I² = 83% | Test for overall effect: Z = 1.21 (P = 0.23) |
| Fosbol et al.  (2020) ^(14)^ | 1.12 [0.92, 1.37] | Heterogeneity: Tau² = 0.10; Chi² = 55.46, df = 19 (P < 0.0001); I² = 66% | Test for overall effect: Z = 1.11 (P = 0.27) |
| Liabeuf et al.  (2020) ^(15)^ | 1.19 [0.92, 1.53] | Heterogeneity: Tau² = 0.19; Chi² = 112.98, df = 19 (P < 0.00001); I² = 83% | Test for overall effect: Z = 1.32 (P = 0.19) |
| Cannata et al.  (2020) ^(16)^ | 1.17 [0.90, 1.51] | Heterogeneity: Tau² = 0.20; Chi² = 114.11, df = 19 (P < 0.00001); I² = 83% | Test for overall effect: Z = 1.16 (P = 0.25) |
| Conversano et al.  (2020) ^(17)^ | 1.14 [0.89, 1.48] | Heterogeneity: Tau² = 0.20; Chi² = 113.05, df = 19 (P < 0.00001); I² = 83% | Test for overall effect: Z = 1.04 (P = 0.30) |
| Felice et al.  (2020) ^(18)^ | 1.24 [0.97, 1.58] | Heterogeneity: Tau² = 0.17; Chi² = 103.60, df = 19 (P < 0.00001); I² = 82% | Test for overall effect: Z = 1.72 (P = 0.09) |
| Tedeschi et al.  (2020) ^(20)^ | 1.21 [0.94, 1.56] | Heterogeneity: Tau² = 0.18; Chi² = 99.58, df = 19 (P < 0.00001); I² = 81% | Test for overall effect: Z = 1.49 (P = 0.14) |
| Jung et al.  (2020) ^(21)^ | 1.11 [0.87, 1.42] | Heterogeneity: Tau² = 0.18; Chi² = 103.77, df = 19 (P < 0.00001); I² = 82% | Test for overall effect: Z = 0.84 (P = 0.40) |
| Bean at al.  (2020) ^(23)^ | 1.17 [0.89, 1.54] | Heterogeneity: Tau² = 0.23; Chi² = 111.70, df = 19 (P < 0.00001); I² = 83% | Test for overall effect: Z = 1.13 (P = 0.26) |
| Mehta et al.  (2020) ^(24)^ | 1.16 [0.90, 1.49] | Heterogeneity: Tau² = 0.19; Chi² = 113.92, df = 19 (P < 0.00001); I² = 83% | Test for overall effect: Z = 1.13 (P = 0.26) |
| Richardson et al.  (2020) ^(26)^ | 1.17 [0.88, 1.54] | Heterogeneity: Tau² = 0.24; Chi² = 111.08, df = 19 (P < 0.00001); I² = 83% | Test for overall effect: Z = 1.09 (P = 0.28) |

**Supp Table 12: Sensitivity analysis from Meta-analysis on COVID-19 mortality comparison between ACEI vs. No ACEI or ARB and ARB vs. No ARB or ACEI**

|  | **ACEI vs. No ACEI or ARB** | | | **ARB vs. no ARB or ACEI** | | |
| --- | --- | --- | --- | --- | --- | --- |
| **Comparison/ Studies excluded** | **Pooled effect size** | **Heterogeneity** | **Statistical significance** | **Pooled effect size** | **Heterogeneity** | **Statistical significance** |
| **Overall effect** | 1.26 [0.89, 1.79] | Heterogeneity: Tau² = 0.18; Chi² = 45.68, df = 9 (P < 0.00001); I² = 80% | Test for overall effect: Z = 1.30 (P = 0.19) | 1.00 [0.71, 1.42] | Heterogeneity: Tau² = 0.15; Chi² = 32.60, df = 8 (P < 0.0001); I² = 75% | Test for overall effect: Z = 0.01 (P = 0.99) |
| **Studies from Asia** | 1.24 [0.95, 1.62] | Heterogeneity: Tau² = 0.00; Chi² = 1.81, df = 4 (P = 0.77); I² = 0% | Test for overall effect: Z = 1.55 (P = 0.12) | 0.80 [0.51, 1.26] | Heterogeneity: Tau² = 0.00; Chi² = 2.64, df = 3 (P = 0.45); I² = 0% | Test for overall effect: Z = 0.97 (P = 0.33) |
| **Studies from the USA** | 1.23 [0.97, 1.56] | Heterogeneity: Not applicable | Test for overall effect: Z = 1.67 (P = 0.09) | 1.15 [0.93, 1.43] | Heterogeneity: Not applicable | Test for overall effect: Z = 1.26 (P = 0.21) |
| **Studies from Europe** | 1.32 [0.66, 2.67] | Heterogeneity: Tau² = 0.45; Chi² = 39.27, df = 3 (P < 0.00001); I² = 92% | Test for overall effect: Z = 0.78 (P = 0.43) | 1.03 [0.55, 1.92] | Heterogeneity: Tau² = 0.33; Chi² = 24.55, df = 3 (P < 0.0001); I² = 88% | Test for overall effect: Z = 0.08 (P = 0.93) |
| **High quality studies**  Gao et al.  (2020) ^(2)^ | 1.00 [0.14, 7.07] | Heterogeneity: Not applicable | Test for overall effect: Z = 0.00 (P = 1.00) | 1.19 [0.38, 3.75] | Heterogeneity: Not applicable | Test for overall effect: Z = 0.29 (P = 0.77) |
| **Leave one out analysis**  **Excluding:** | | | | | | |
| Gao et al.  (2020) ^(2)^ | 1.27 [0.89, 1.81] | Heterogeneity: Tau² = 0.19; Chi² = 45.55, df = 8 (P < 0.00001); I² = 82% | Test for overall effect: Z = 1.30 (P = 0.19) | 0.99 [0.68, 1.43] | Heterogeneity: Tau² = 0.16; Chi² = 32.59, df = 7 (P < 0.0001); I² = 79% | Test for overall effect: Z = 0.08 (P = 0.94) |
| Li & Wang et al.  (2020) ^(6)^ | 1.31 [0.91, 1.90] | Heterogeneity: Tau² = 0.19; Chi² = 43.79, df = 8 (P < 0.00001); I² = 82% | Test for overall effect: Z = 1.43 (P = 0.15) | 1.04 [0.71, 1.52] | Heterogeneity: Tau² = 0.16; Chi² = 29.57, df = 7 (P = 0.0001); I² = 76% | Test for overall effect: Z = 0.19 (P = 0.85) |
| Meng et al.  (2020) ^(8)^ | 1.25 [0.87, 1.78] | Heterogeneity: Tau² = 0.19; Chi² = 45.47, df = 8 (P < 0.00001); I² = 82% | Test for overall effect: Z = 1.22 (P = 0.22) | 1.01 [0.71, 1.44] | Heterogeneity: Tau² = 0.16; Chi² = 32.32, df = 7 (P < 0.0001); I² = 78% | Test for overall effect: Z = 0.04 (P = 0.97) |
| Wang et al.  (2020) ^(10)^ | 1.24 [0.81, 1.89] | Heterogeneity: Tau² = 0.24; Chi² = 45.45, df = 8 (P < 0.00001); I² = 82% | Test for overall effect: Z = 1.00 (P = 0.32) | NR | NR | NR |
| Fosbol et al.  (2020) ^(14)^ | 1.13 [0.87, 1.46] | Heterogeneity: Tau² = 0.06; Chi² = 15.99, df = 8 (P = 0.04); I² = 50% | Test for overall effect: Z = 0.91 (P = 0.37) | 0.91 [0.70, 1.17] | Heterogeneity: Tau² = 0.04; Chi² = 10.27, df = 7 (P = 0.17); I² = 32% | Test for overall effect: Z = 0.76 (P = 0.45) |
| Conversano et al.  (2020) ^(17)^ | 1.16 [0.80, 1.68] | Heterogeneity: Tau² = 0.19; Chi² = 42.52, df = 8 (P < 0.00001); I² = 81% | Test for overall effect: Z = 0.77 (P = 0.44) | 0.97 [0.66, 1.42] | Heterogeneity: Tau² = 0.17; Chi² = 32.60, df = 7 (P < 0.0001); I² = 79% | Test for overall effect: Z = 0.16 (P = 0.87) |
| Felice et al.  (2020) ^(18)^ | 1.38 [0.97, 1.96] | Heterogeneity: Tau² = 0.16; Chi² = 39.18, df = 8 (P < 0.00001); I² = 80% | Test for overall effect: Z = 1.80 (P = 0.07) | 1.10 [0.78, 1.55] | Heterogeneity: Tau² = 0.13; Chi² = 26.41, df = 7 (P = 0.0004); I² = 73% | Test for overall effect: Z = 0.55 (P = 0.58) |
| Tedeschi et al.  (2020) ^(20)^ | 1.36 [0.95, 1.95] | Heterogeneity: Tau² = 0.16; Chi² = 32.50, df = 8 (P < 0.0001); I² = 75% | Test for overall effect: Z = 1.70 (P = 0.09) | 1.04 [0.70, 1.54] | Heterogeneity: Tau² = 0.16; Chi² = 26.33, df = 7 (P = 0.0004); I² = 73% | Test for overall effect: Z = 0.20 (P = 0.84) |
| Richardson et al.  (2020) ^(26)^ | 1.25 [0.81, 1.93] | Heterogeneity: Tau² = 0.26; Chi² = 43.72, df = 8 (P < 0.00001); I² = 82% | Test for overall effect: Z = 1.02 (P = 0.31) | 0.93 [0.58, 1.51] | Heterogeneity: Tau² = 0.28; Chi² = 32.03, df = 7 (P < 0.0001); I² = 78% | Test for overall effect: Z = 0.29 (P = 0.77) |
| Tan et al.  (2020) ^(9)^ | 1.27 [0.89, 1.82] | Heterogeneity: Tau² = 0.19; Chi² = 45.28, df = 8 (P < 0.00001); I² = 82% | Test for overall effect: Z = 1.34 (P = 0.18) | 1.04 [0.74, 1.46] | Heterogeneity: Tau² = 0.14; Chi² = 29.59, df = 7 (P = 0.0001); I² = 76% | Test for overall effect: Z = 0.21 (P = 0.83) |

**Supp Table 13:** **Sensitivity analysis from Meta-analysis on mortality from COVID-19 in people with a history of hypertension**

| **Comparison** | **Pooled effect size** | **Heterogeneity** | **Statistical significance** |
| --- | --- | --- | --- |
| **Overall effect** | 0.72 [0.52, 0.99] | Heterogeneity: Tau² = 0.10; Chi² = 23.97, df = 10 (P = 0.008); I² = 58% | Test for overall effect: Z = 2.03 (P = 0.04) |
| **Studies from Asia** | 0.60 [0.42, 0.88] | Heterogeneity: Tau² = 0.01; Chi² = 7.17, df = 7 (P = 0.41); I² = 2% | Test for overall effect: Z = 2.65 (P = 0.008) |
| **Studies from the USA** | 1.18 [0.99, 1.41] | Heterogeneity: Not applicable | Test for overall effect: Z = 1.84 (P = 0.07) |
| **Studies from Europe** | 0.71 [0.45, 1.12] | Heterogeneity: Tau² = 0.06; Chi² = 2.17, df = 1 (P = 0.14); I² = 54% | Test for overall effect: Z = 1.49 (P = 0.14) |
| **Removing possible dual reporting in multiple studies** | | | |
| **Excluding:**  Huang et al.  (2020) ^(5)^  Zhang et al.  (2020) ^(12)^ | 0.81 [0.60, 1.09] | Heterogeneity: Tau² = 0.07; Chi² = 17.00, df = 8 (P = 0.03); I² = 53% | Test for overall effect: Z = 1.40 (P = 0.16) |
| **Leave one out analysis**  **Excluding:** | | | |
| Hu et al.  (2020) ^(4)^ | 0.70 [0.51, 0.97] | Heterogeneity: Tau² = 0.10; Chi² = 23.27, df = 9 (P = 0.006); I² = 61% | Test for overall effect: Z = 2.12 (P = 0.03) |
| Huang et al.  (2020) ^(5)^ | 0.72 [0.52, 1.00] | Heterogeneity: Tau² = 0.11; Chi² = 23.34, df = 9 (P = 0.005); I² = 61% | Test for overall effect: Z = 1.96 (P = 0.05) |
| Li & Wang et al.  (2020) ^(6)^ | 0.68 [0.47, 1.00] | Heterogeneity: Tau² = 0.13; Chi² = 23.44, df = 9 (P = 0.005); I² = 62% | Test for overall effect: Z = 1.97 (P = 0.05) |
| Meng et al.  (2020) ^(8)^ | 0.72 [0.52, 1.00] | Heterogeneity: Tau² = 0.11; Chi² = 23.78, df = 9 (P = 0.005); I² = 62% | Test for overall effect: Z = 1.99 (P = 0.05) |
| Tan et al.  (2020) ^(9)^ | 0.75 [0.55, 1.01] | Heterogeneity: Tau² = 0.09; Chi² = 21.08, df = 9 (P = 0.01); I² = 57% | Test for overall effect: Z = 1.88 (P = 0.06) |
| Yang et al.  (2020) ^(11)^ | 0.74 [0.54, 1.02] | Heterogeneity: Tau² = 0.10; Chi² = 22.05, df = 9 (P = 0.009); I² = 59% | Test for overall effect: Z = 1.81 (P = 0.07) |
| Zhang et al.  (2020) ^(12)^ | 0.80 [0.60, 1.08] | Heterogeneity: Tau² = 0.07; Chi² = 17.64, df = 9 (P = 0.04); I² = 49% | Test for overall effect: Z = 1.47 (P = 0.14) |
| Zhou et al.  (2020) ^(13)^ | 0.72 [0.52, 1.01] | Heterogeneity: Tau² = 0.11; Chi² = 23.47, df = 9 (P = 0.005); I² = 62% | Test for overall effect: Z = 1.93 (P = 0.05) |
| Felice et al.  (2020) ^(18)^ | 0.77 [0.55, 1.07] | Heterogeneity: Tau² = 0.09; Chi² = 19.75, df = 9 (P = 0.02); I² = 54% | Test for overall effect: Z = 1.58 (P = 0.11) |
| Tedeschi et al.  (2020) ^(20)^ | 0.64 [0.41, 1.00] | Heterogeneity: Tau² = 0.20; Chi² = 23.20, df = 9 (P = 0.006); I² = 61% | Test for overall effect: Z = 1.95 (P = 0.05) |
| Richardson et al.  (2020) ^(26)^ | 0.66 [0.50, 0.87] | Heterogeneity: Tau² = 0.03; Chi² = 10.88, df = 9 (P = 0.28); I² = 17% | Test for overall effect: Z = 2.96 (P = 0.003) |
