# Supplementary material for: Meta-analysis of the association between angiotensin pathway inhibitors and COVID-19 severity and mortality
Source: Syst Rev. 2021 Sep 7;10:243. doi: 10.1186/s13643-021-01802-6 (PMC8421238; doi:10.1186/s13643-021-01802-6)

**S2: Additional Plots and Figures**

**List of Supplementary Figures**

Supp Fig 1: Forest plot of COVID-19 severity and association with prescription of ACEI

Supp Fig 2: Forest plot of COVID-19 severity and association with prescription of ARB.

Supp Fig 3: Forest plot of COVID-19 severity and association with prescription of ACEI/ARB in people with a previous history of hypertension.

Supp Fig 4: Funnel plot of COVID-19 severity (left) and mortality (right) and association with prescription of ACEI/ARB in people with a previous history of hypertension.

Supp Fig 5: Funnel plot of COVID-19 severity (left) and mortality (right) and association with prescription of ACEI

Supp Fig 6: Funnel plot of COVID-19 severity (left) and mortality (right) and association with prescription of ARB

Supp Fig 7: Funnel plot of COVID-19 severity (left) and mortality (right) and association with prescription of ACEI/ARB in people with a prior history of hypertension

Supp Fig 8: Forest plot of COVID-19 mortality and association with prescription of ACEI

Supp Fig 9: Forest plot of COVID-19 mortality and association with prescription of ARB


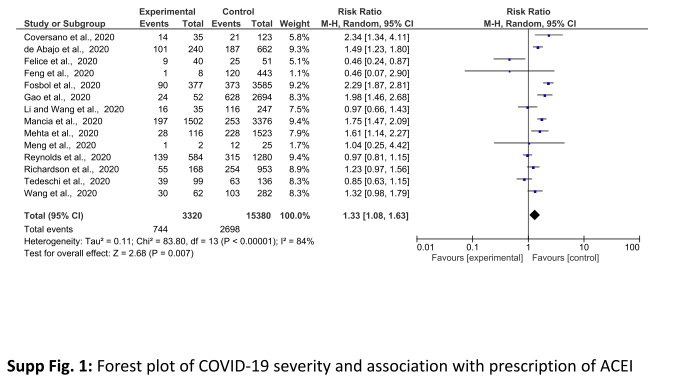


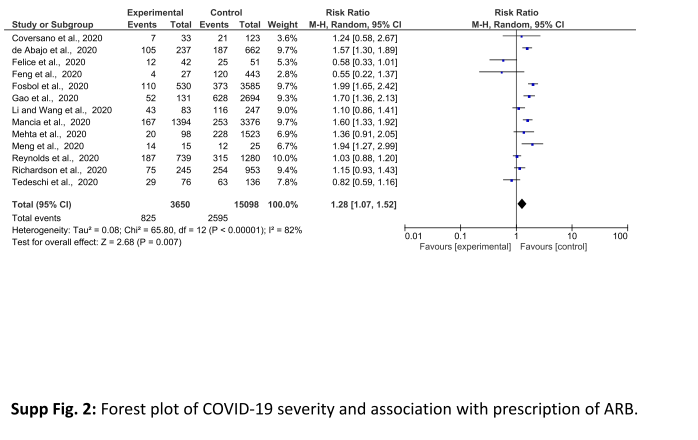


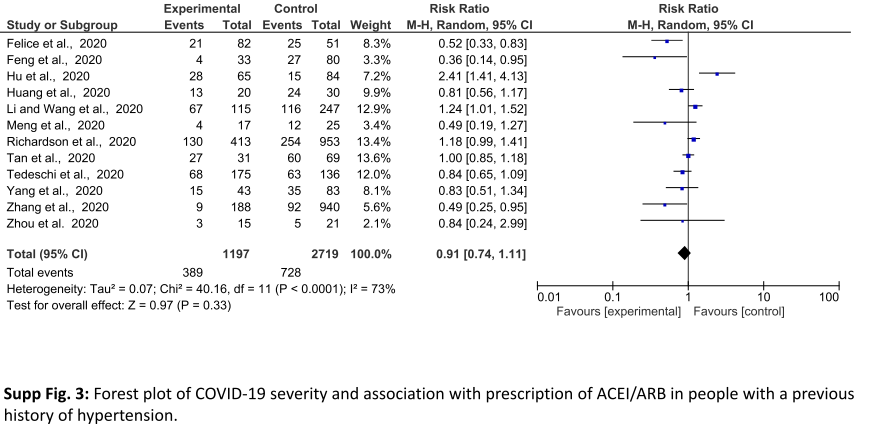


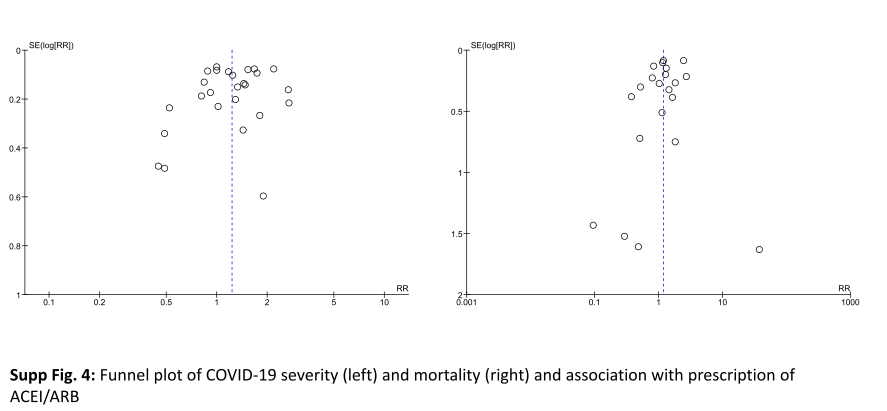


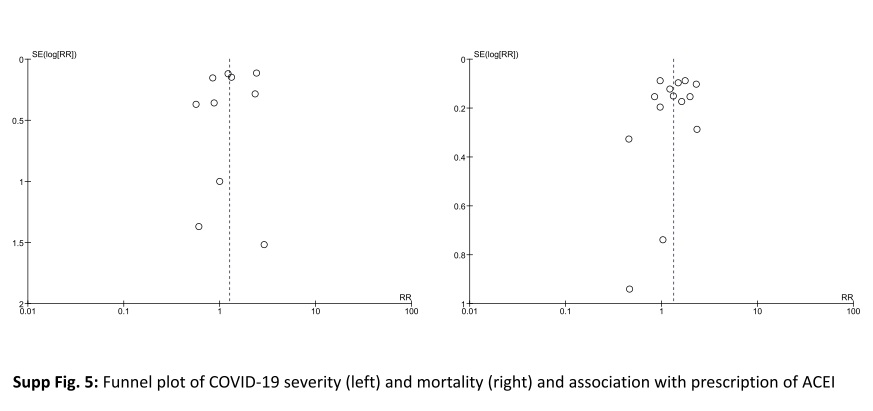


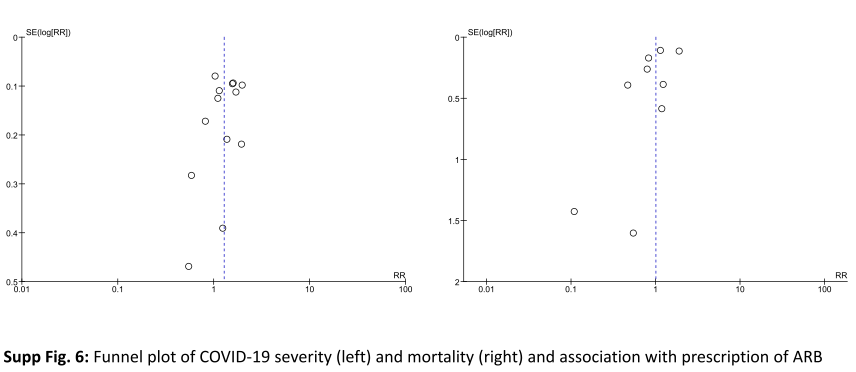


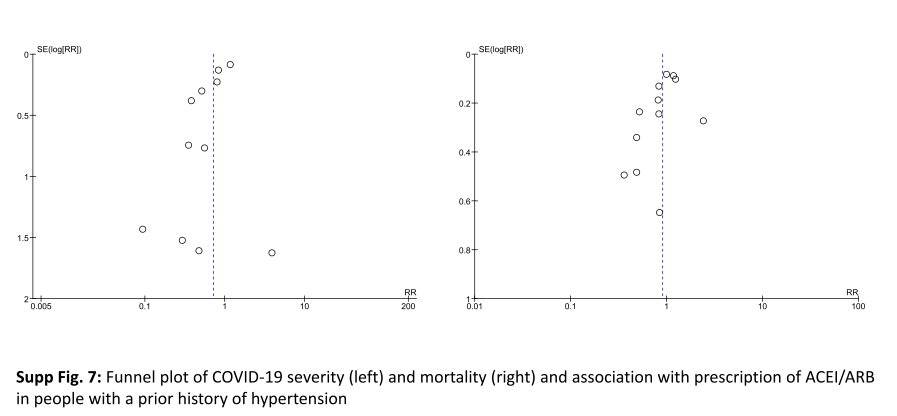


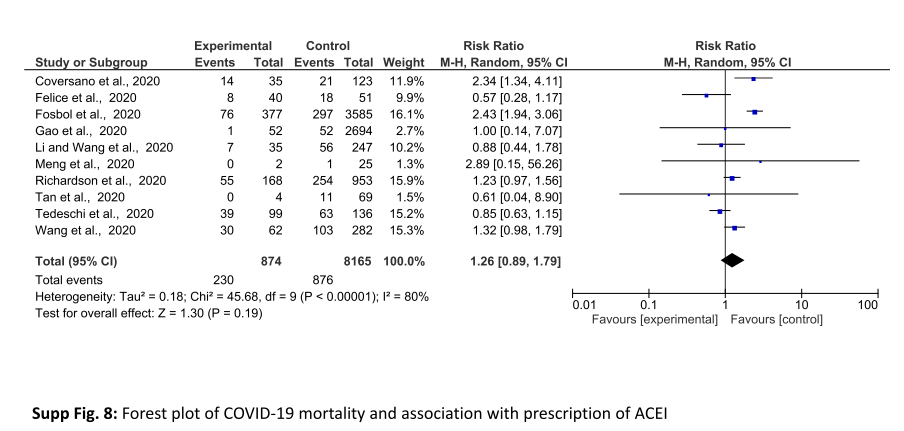


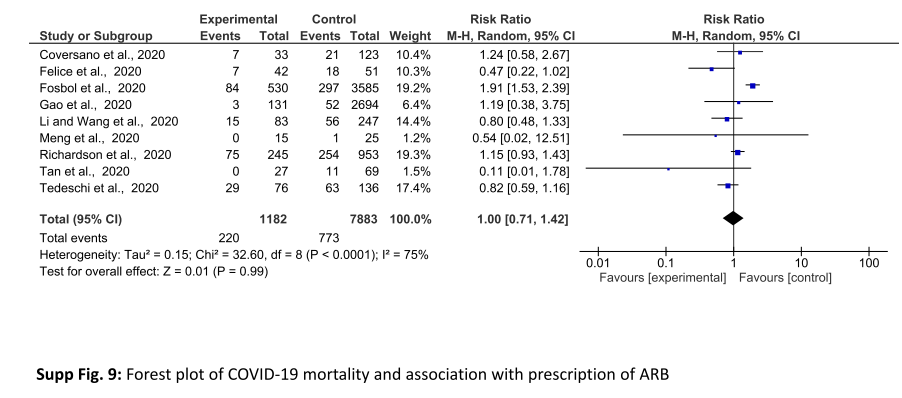


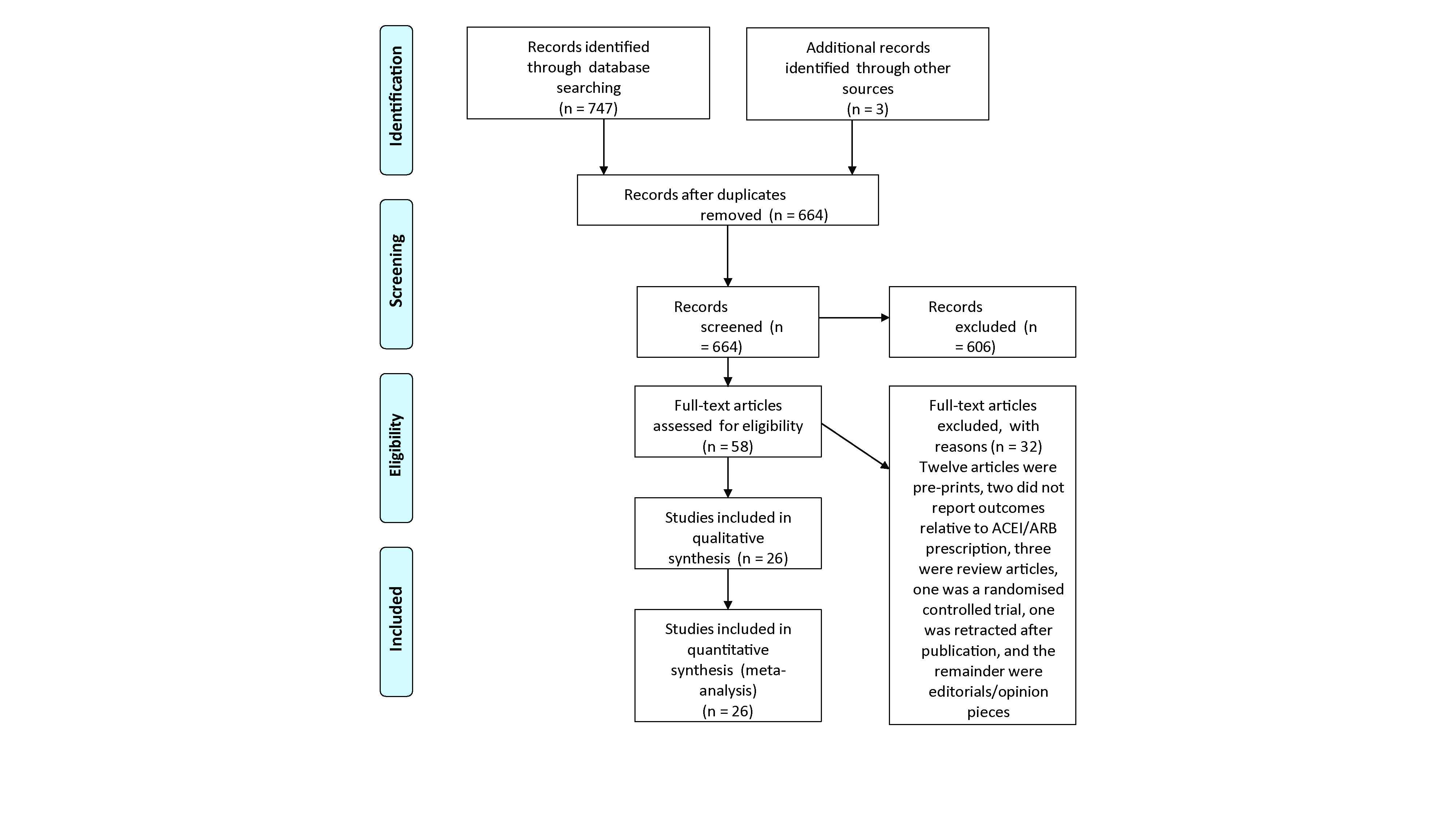


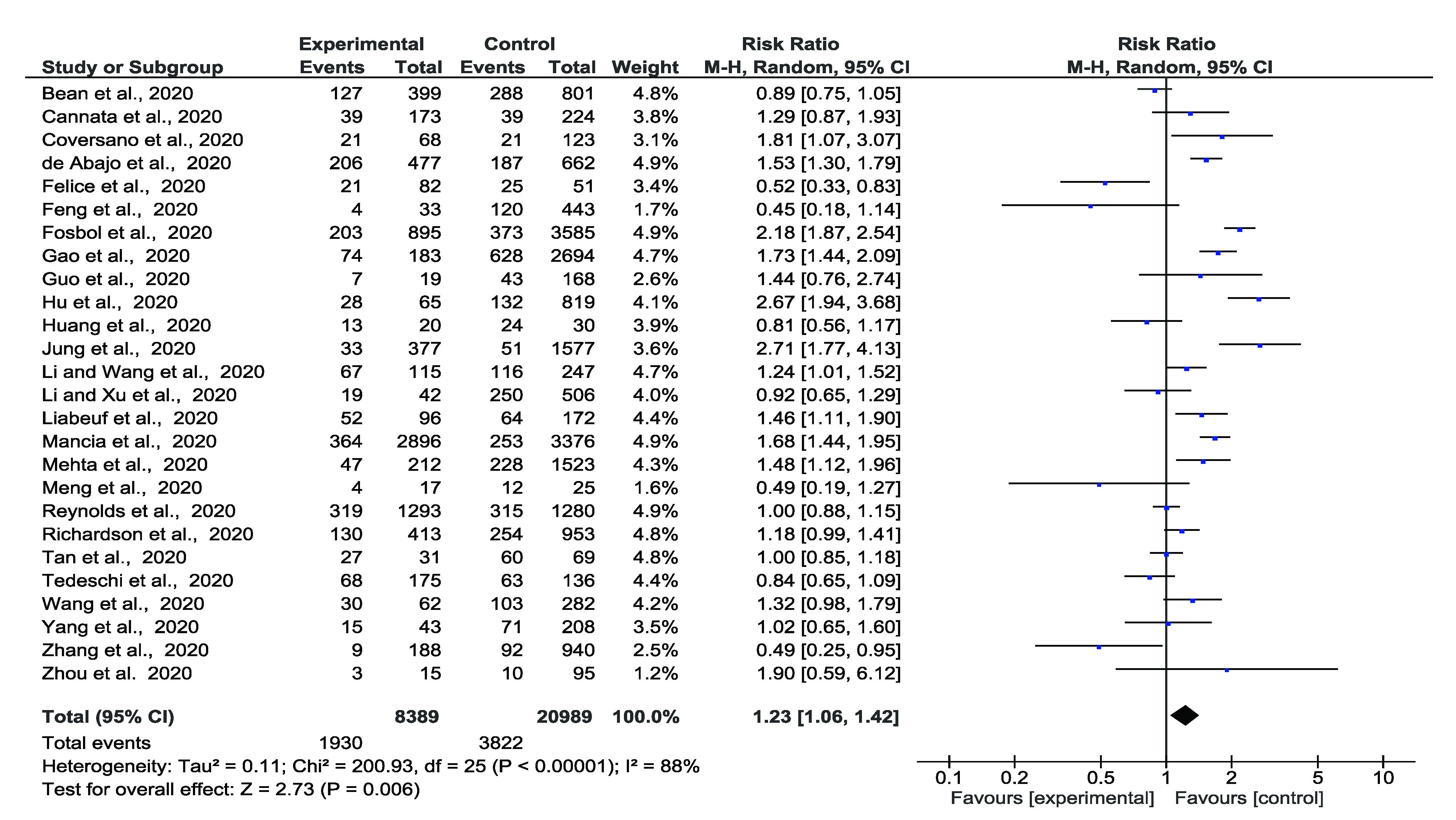


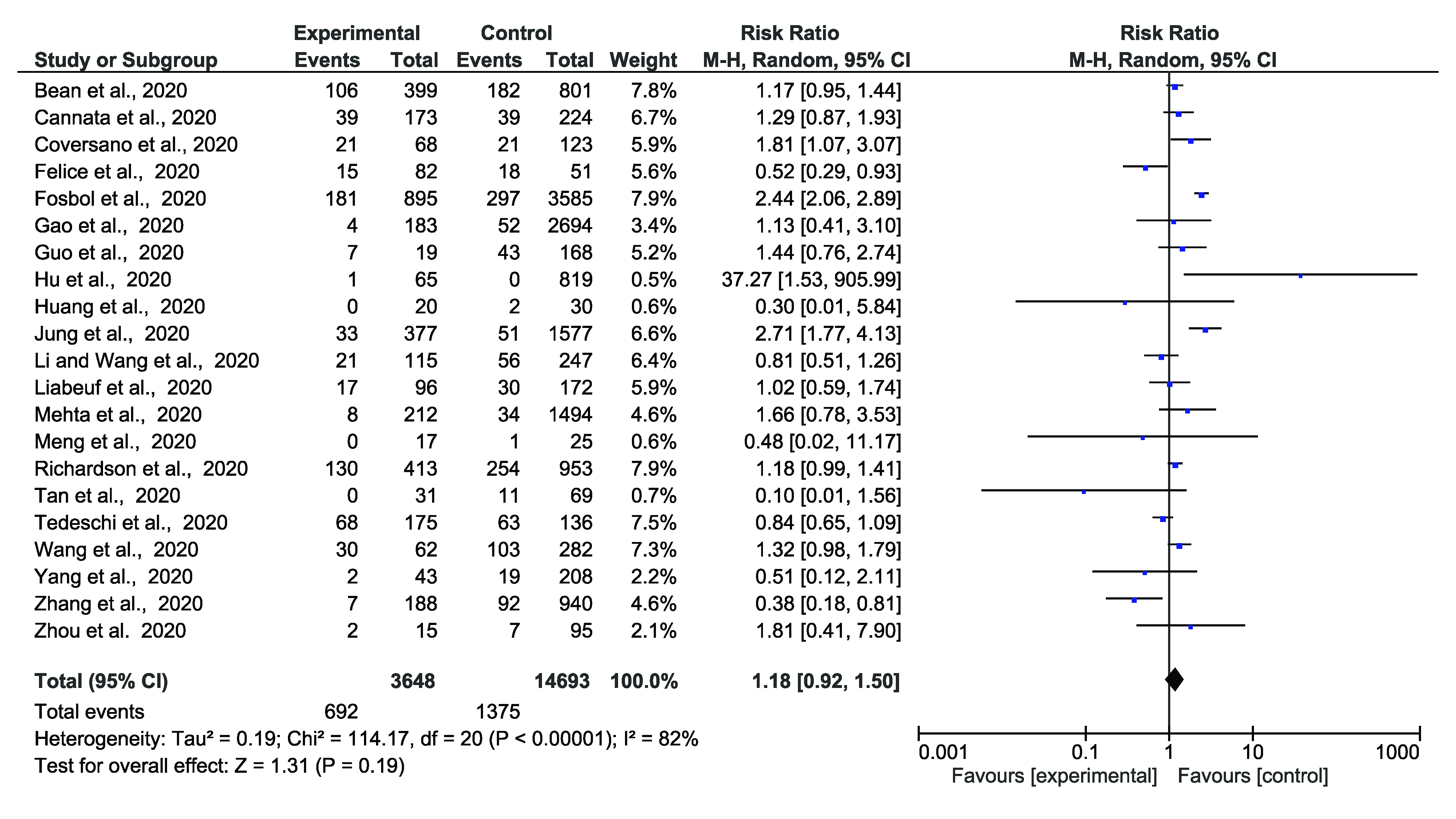


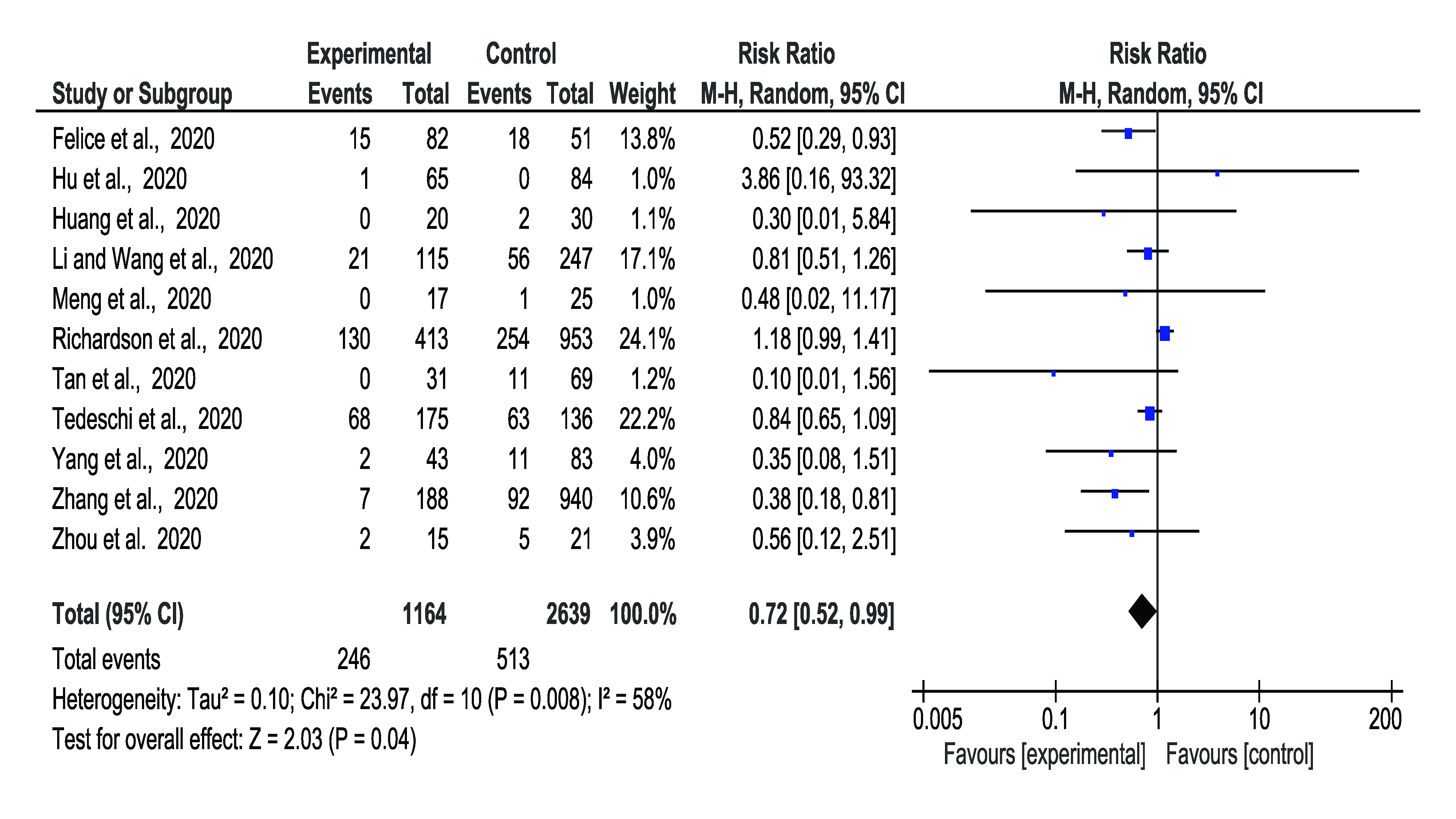

Supplement: Supplementary file 2 — Additional file 2: S2: Additional Plots and Figures. [file 13643_2021_1802_MOESM2_ESM.docx]
